# CBMNet: a dual-attention enhanced ConvNeXt model for accurate G.V. Black type I–III classification in intraoral periapical radiographs

**DOI:** 10.1038/s41598-025-22975-3

**Published:** 2025-11-10

**Authors:** Aneetta Joy Parathanath, Manimaran A

**Affiliations:** Department of Mathematics, School of Advanced Sciences, VIT-AP University, Amaravati, Andhra Pradesh 522237 India

**Keywords:** Dental caries, ConvNeXt, CBAM, MSAM, Particle swarm optimization, StyleGAN2-ADA, Intraoral periapical radiographs, Deep learning, Attention mechanisms, Dental diagnostics, Computational biology and bioinformatics, Diseases, Health care, Mathematics and computing, Medical research

## Abstract

Dental caries is among the most prevalent oral diseases worldwide, and accurate radiographic detection remains a clinical challenge, particularly for lesions defined by the G.V. Black classification. This study aimed to develop and evaluate CBMNet, a dual-attention enhanced ConvNeXt-Tiny model, for automated classification of G.V. Black Classes I-III using intraoral periapical radiographs. A total of 1103 anonymized periapical radiographs were retrospectively collected from the Sibar Institute of Dental Sciences, India, covering G.V. Black Class I (*n* = 408), Class II (*n* = 490), and Class III (*n* = 205). To address class imbalance, minority classes were supplemented with high-fidelity synthetic images generated via StyleGAN2-ADA, validated using BRISQUE scores and blinded expert review. Images were pre-processed with CLAHE and median filtering, and CBMNet was implemented by integrating Convolutional Block Attention Module (CBAM) and Multi-Scale Attention Module (MSAM) into a ConvNeXt-Tiny backbone. Hyperparameters were optimized using Particle Swarm Optimization (PSO). Performance was evaluated through stratified 5-fold cross-validation, ablation studies, and a held-out real-image test set, with additional robustness testing via test-time augmentation(TTA). CBMNet achieved a mean validation accuracy of 93.26% ( 0.81)across folds and a final held-out test accuracy of 92% with TTA. Class-wise evaluation showed high precision (Class I:0.90, Class II:0.87, Class III:0.99), recall (Class I:0.94, Class II:0.90, Class III:0.91), and F1-scores (Class I:0.92, Class II:0.89, Class III:0.95). Ablation analysis confirmed the complementary contributions of CBAM, MSAM, and TTA. Compared with baseline models (ResNet50, EfficientnetB0, DenseNet121), CBMNet consistently outperformed in overall and class-specific metrics. The proposed CBMNet framework demonstrated robust diagnostic performance for automated classification of G.V. Black Classes I-III from periapical radiographs, with accuracy and class-wise metrics exceeding 90%. By integrating dual-attention mechanisms, GAN-based augmentation, and PSO-driven optimization, CBMNet provides a reliable, interpretable, and clinically relevant tool that may support early detection and standardized diagnosis of dental caries. Future studies with multi-centre datasets and prospective clinician comparisons are warranted to further validate clinical applicability.

## Introduction

Dental caries is recognized as one of the most pervasive chronic conditions on a global scale^1^. Teeth are vital for grinding, chewing and breaking down food to aid in swallowing and digestion. Dental cavities, commonly known as tooth decay or caries, are a globally prevalent dental issue. This infectious disease can impact individuals of any age^2^. This microbial illness is typified by the demineralization and breakdown of the teeth’s hard structures, frequently resulting in cavitation. It is a result of bacterial fermentation of food debris that produces acid^3^. Dental caries development is influenced by factors like sugar consumption, saliva, and fluoride exposure. It can occur on any tooth surface but is more prevalent in protected areas where biofilm matures, such as pits, grooves, fissures along the gingival margin^4^. Accurate diagnosis and disease detection are critical priorities in dentistry and related biomedical disciplines^5^.

Manually identifying cavities is a difficult task for dentists, as it demands the examination of each tooth individually. The 4th Chinese National Oral Health Epidemiological Survey found that dental caries is the most common oral health problem, affecting 70.81% of kids aged 3 to 5. The incidence goes up progressively with age, reaching 80.7% in people aged 65 to 74^6^. This problem might be worse in places with expensive dental care which is further not covered under the insurance plans. Though X-rays are the main way to find cavities, dentists need to carefully look at each tooth on the X-rays to see how worse the issue is, find cavities, and identify the ways of treatment. Even trained professionals sometimes miss small cavities, leading to wrong diagnosis or further spreading. Moreover, different dentists may have different ideas on what a carious tooth looks like; one claiming it as carious while another as healthy. Teeth have to be handled with utmost care. If not, diseases like caries can cause teeth to fall out for good. Finding cavities in their early stages is hard because they are commonly missed. A dependable mechanism for early detection is quite important to deal with this^7^.

G. V. Black, also known as “The Father of Modern Dentistry,” played a big role in making dentistry a separate profession. One of his most important achievements was coming up with a way to group carious lesions based on where they are on the tooth surface. This approach put cavities into classes I through V, with some having more than one surface combination. At first, decay on the tips of anterior teeth and the cusps of posterior teeth was not included, but it was later added as class VI. This procedure is still commonly used since it gives dental students and experts a way to find cavities and figure out the best way to treat them. The classification also helps choose the right restorative materials for the area that needs repair, which makes sure that the management is effective^8^. G.V. Black’s basic classification system from 1908 put carious lesions into five classes based on the type of tooth and the specific surfaces that were affected. Each lesion had its own unique cavity configuration. According to Black, it was very important to remove all afflicted dentine and make cavities bigger so that they reach places that can clean themselves. This will stop decay from happening again.

- *Class I*: Lesions located in pits and fissures of the occlusal third of molars and premolars, the occlusal two-thirds of molars and premolars, and the lingual surfaces of anterior teeth.
- *Class II*: Lesions on the proximal surfaces of molars and premolars.
- *Class III*: Lesions on the proximal surfaces of anterior teeth, excluding the incisal angles.
- *Class IV*: Lesions on the proximal surfaces of anterior teeth, including the incisal angles.
- *Class V*: Lesions affecting the gingival third of the facial or lingual surfaces of both anterior and posterior teeth.

In 1956, Simon added another group to Black’s plan. Class VI contains cavities that occur on the edges of the incisors or the cusps of the molars. Mechanical wear, chemical dissolution, or natural wear on the tooth surface are the most common causes of these cavities^9^. This technique makes it easy to sort carious lesions by where they are and how serious they are, which helps dentists locate and diagnose dental decay in a more organized fashion. Black’s method gives dentists a common language that makes it easier for doctors, teachers, and students to discuss about how to treat cavities. By grouping cavities based on its type and location, the categorization helps find the best strategy to fix them. Class IV lesions that harm the incisal angles need to be treated differently from Class I lesions that are in pits and fissures. It’s still a good idea to think about how to make cavities go into regions that clean themselves. It helps maintain restorations clean and in good shape for a long time, which minimizes the risk of acquiring cavities again.

Visual and tactile exams frequently find caries lesions, but imaging methods including intraoral scanning, optical coherence tomography, quantitative light-induced fluorescence, radiography, and near-infrared light transillumination can help make the diagnosis more accurate. Even if imaging methods have improved a lot, it is still challenging to look at the pictures and find cavities. Dentists often miss a lot of early caries lesions in different methods of imaging, such radiography, and their diagnosis accuracy and treatment choices are very diverse. Finding early caries with imaging is quite tricky because it is not very sensitive and people may have various results. Some people think that deep learning might be able to help with these issues^10^. Dentists can now tell how serious a cavity is through new computer techniques and technologies. Tele-dentistry allows patients get dental care from far away. It also makes it easier to discover cavities at different stages by employing neural networks and tools that are connected to the internet^11^. Artificial Intelligence (AI) has gotten a lot more complicated and powerful, especially in the medical industry. AI is changing the game in dentistry by making predictive machine learning models better, which helps doctors take better care of their patients. In dental practice, it helps dentists make correct diagnoses, plan treatments, and detect oral health problems early, which leads to better outcomes. Most of the time, dental diagnostics employ pictures of the mouth from multiple perspectives. A person has to look at X-rays by hand to see if there are bone loss, cavities, or other dental features. There are automated ways to diagnose dental disorders, but there are still issues including small datasets, class imbalance, limited generalizability, and a lack of transparency and interpretability. They are not utilized in clinical practice as much either because they have not been evaluated enough by independent sources. Even if they show potential^12^, machine learning and deep learning techniques are not yet fully applied in conventional dentistry.

It is hard for typical image processing to find features, especially when it comes to complicated categorization. Deep learning, on the other hand, has figured out how to get past these problems by learning features straight from raw data. While CNNs and vision transformers have demonstrated strong performance in medical imaging^13–15^ and GANs have been used to expand limited datasets^16,17^ their role in clinical dentistry must be considered in terms of patient impact rather than only technical gains. Early lesions missed on radiographs can progress to pulpal involvement, requiring root canal treatment or extraction; false positives may lead to unnecessary restorative procedures and irreversible loss of healthy tooth structure. Moreover, diagnostic variability among clinicians means that the same radiograph may lead to different interpretations and treatment plans, highlighting the need for standardized, reproducible decision support.

From a computational perspective, limited dataset size and class imbalance remain critical barriers. Class III anterior proximal lesions are relatively rare both clinically and in radiographic archives, and their underrepresentation can bias models towards more common lesion types. This reproduces a well-known clinical blind spot, since anterior caries are often among the most overlooked in practice. Likewise, small single-centre datasets risk overfitting, raising the possibility of poor generalization and compromised patient outcomes. Addressing these barriers requires augmentation strategies that expand underrepresented classes while preserving morphological fidelity, and model designs that can localize subtle radiographic changes linked to early caries.

The primary objective of this study is therefore to develop an automated, reliable, and clinically applicable system for classifying dental caries in intraoral periapical radiographs, focusing on Class I, II, and III lesions from the G.V. Black classification. To address key limitations in existing approaches – such as data scarcity, class imbalance, and diagnostic inconsistency – we introduce a hybrid visual learning framework. This integrates StyleGAN2-ADA for high-fidelity synthetic image generation with a novel dual-attention classifier, CBMNet, which incorporates optimized hyperparameters, advanced regularization, and perceptual quality filtering. By linking technical advances directly with clinical priorities, CBMNet is designed not only for classification performance but also for improving early diagnosis, inter-clinician consistency, and reliability in treatment planning. The rest of the sections are set up like this:

- The section "Existing work" gives an overview of the current research on how deep learning can be used to find dental cavities.
- The section "Materials and methods" goes into detail about the materials and methods, such as how data was collected, how synthetic images were made, and the proposed framework.
- The section "Experimental configuration" talks about how the experiment was set up, including how the data was pre-processed, how the classifiers were trained, and how the evaluations were done.
- The section "Results" talks about the results, such as performance metrics and how the model acted across different types of caries.
- The section "Discussion" wraps up the paper with a review of the results and ideas for further research.

## Existing work

Artificial intelligence has shown strong potential in dental imaging, but challenges such as small datasets, class imbalance, and lack of standardized validation continue to limit clinical translation^18^. Below, we summarize key prior works, their contributions, and limitations, before highlighting how CBMNet addresses unresolved gaps.

Sornam and Prabhakaran^19^ combined linear adaptive particle swarm optimization with a back-propagation neural network on 120 periapical radiographs, achieving 99.16% accuracy. However, the small dataset restricts generalizability. Agulan et al.^20^employed YOLOv8 model for G.V. Black classification on a small dataset sourced from Roboflow expanded through augmentation, reaching 95.1% mAP50, but the model struggled to detect multiple lesions in a single tooth. Patil et al^21^. used a wavelet-statistical feature approach with an Adaptive Dragonfly Algorithm-trained neural network, outperforming conventional classifiers but constrained by dataset size.

Priya et al.^22^, combined DenseUNet + + with a ViT-based Multiscale Residual Dense Net with GRU (ViT-MRDGRU) classifier for segmentation and classification on panoramic datasets, yielding 96.59% accuracy but with computational cost and potential overfitting. Singh and Sehgal^23^applied a CNN-LSTM optimized by Dragonfly for G.V. Black classification on 1500 periapical images, achieving 96% accuracy but lacking external validation. Chen et al.^24^, integrated Mask R-CNN with a modified U-Net for panoramic radiographs, achieving high sensitivity but facing difficulty with narrow-tooth structures. Hossain, Md Shakhawat, et al.^25^ introduced CaViT, a ViT-based tool for smartphone images, achieving 95.3% accuracy for early-stage caries, but the method was limited to single-tooth inputs. Imak et al.^26^ designed a multi-input CNN ensemble for 340 periapical images, achieving 991.13% accuracy, though validation across populations was absent. Esmaeilyfard et al.^27^ classified CBCT images with a multi-input CNN (785 scans), obtaining 95.3% accuracy, but performance dropped for cervical caries due to insufficient data.

Hasnain et al.^28^ demonstrated EfficientNet-B5 on 549 panoramic radiographs, achieving 98.23% accuracy, but again with limited dataset diversity. Salunke et al.^29^ trained a CNN on 1336 RVG images, reporting 94.2% accuracy, though generalization to other imaging systems was not tested. Liu et al.^30^applied CNNs to 188 augmented X-rays, achieving 99.5% accuracy with DenseNet-121, but the small original dataset raised concerns about robustness. They also validated CNNs for lesion classification, again highlighting reliance on limited augmented datasets. Beyond classification, recent advances in generative AI are transforming dentistry. For example, Ahmet et al^31^. demonstrated generative AI applications in dental education and clinical decision support, while Salbas et al.^32^ showed its use in exam evaluation and standardization. These works highlight the breadth of AI in dentistry, though most remain outside structured G.V. Black classification tasks.

In summary, while prior studies report high accuracies, most are constrained by small, imbalanced datasets, limited lesion diversity, and lack of clinical validation. CBMNet addresses these gaps by focusing on G.V. Black Classes I-III, mitigating imbalance with GAN augmentation validated by experts, enhancing lesion localization through dual attention, and ensuring reproducibility via PSO optimization and test-time augmentation. This positions CBMNet as both a technical and clinically relevant advancement in automated caries classification.

## Materials and methods

### Data collection

An automated G.V. Black caries classification system will only work well if it is trained on different types of data. We got the dataset for this work from the SIBAR Institute of Dental Sciences in Guntur, India. A digital radiography system is used to take the intraoral periapical radiographs. This system is made to make sure that the images are high-resolution and have as little noise and artifacts as possible. The X-Mind DC dental X-ray device (Aceton, France) and the Vista Scan Mini Easy PSP scanner (Durr Dental, Germany) work together to take radiographs. The system used Size 2 photostimulable phosphor (PSP) plates and exposure periods that worked best were between 0.12 and 0.32 s. Scanning the plates at 20-line pairs per millimetre (lp/mm) gives a clear picture. The Vista scan imaging software processes and extracts digital images, which are then saved in PNG format. This makes them good for AI-based classification tasks and more analysis. The original G.V. Black categorization system divides caries into six main classes. Our study solely looks at Class I, Class II, and Class III. The reason for this selective approach is that Class IV (incisal edge caries), Class V (cervical caries), and Class VI (cusp tip caries) can all be seen in a clinical setting without the need for X-rays. Radiographs are not the main way to diagnose these lesions; instead, they are diagnosed by looking at and feeling them. Patients usually only go to the doctor after their cavities have gotten really bad, which means they need X-rays to check out the deep lesions. Periapical radiographs are often used to find Class I (occlusal), Class II (proximal posterior), and Class III (proximal anterior) caries since they are harder to see with the naked eye but need to be treated right away. Most clinical cases that need to be looked at with X-rays are in Class I, II, or III. By focusing on these three classifications, we make sure that automated categorization algorithms are useful in real-life clinical settings. So, the goal of our study is to improve the ability to diagnose caries that can be seen on X-rays in real time, while leaving out cases that can be easily found by a clinical exam alone.

To maintain the data quality and diagnostic relevance high, strict rules were applied to choose which datasets could and could not be included.

Criteria for Inclusion.

- X-rays that indicate at least one cavity that can be seen.
- High-resolution pictures to make sure the diagnosis is obvious.
- Dentists used G. V. Black’s Class I-III categorization system to group cavities.

Criteria for Not Being Included.

- Images that are grainy, distorted, or have low contrast, which could make it tougher for automatic feature extraction to perform.
- X-rays that show braces, crowns, or metal restorations on teeth, which could make it tougher to figure out what type of teeth they are.
- Teeth that are very close together or other things that make it hard to see cavities.
- X-rays that show more than one oral issue, like fractures, cysts, or periodontal disease, which makes it hard to put them in a category.

**Fig. 1:**
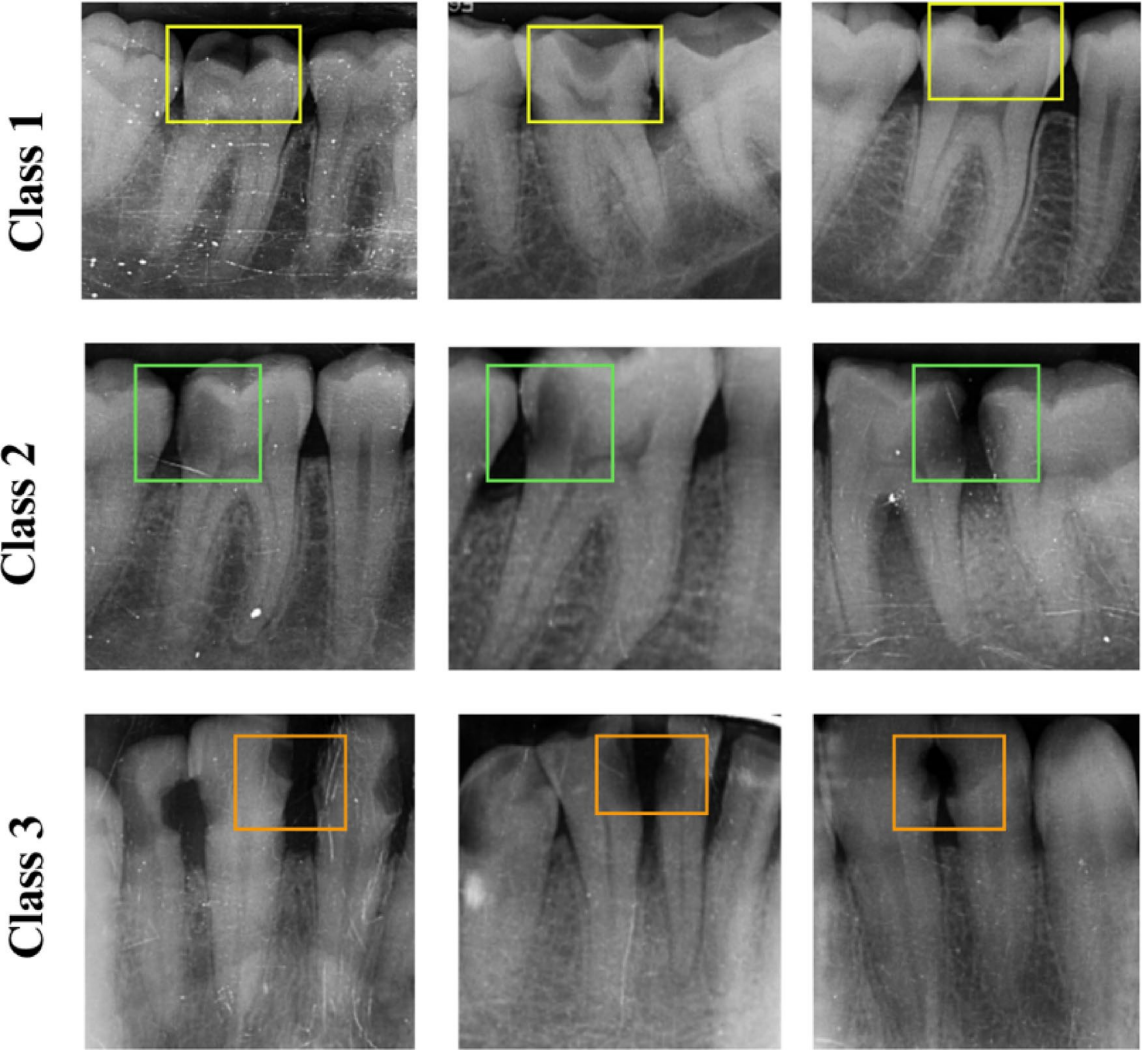
Sample intraoral periapical radiographs depicting G.V. Black Class I–III caries, with carious regions highlighted by bounding boxes.

After applying inclusion and exclusion criteria, a total of 1103 intraoral periapical radiographs are selected. Representative intraoral periapical radiographs collected for this study are shown in Fig. 1, covering the range of clinical variability across the caries classes. It should be noted that the age range of 18–65 years occurred incidentally, as all eligible caries-positive radiographs available in the institutional archive during data collection happened to fall within this range. These images are categorized into the three G.V. Black classes as shown in Table 1:

**Table 1:** Class-wise distribution of images collected across the three diagnostic categories.

Caries Class(G. V. Black)	Description	Number of images
Class I	Occlusal caries on premolars/molars	408
Class II	Proximal caries in posterior teeth	490
Class III	Proximal caries in anterior teeth	205

During data analysis, it is observed that Class III had significantly fewer samples compared to Class I and Class II, leading to a class imbalance problem. To mitigate this, synthetic image generation using StyleGAN2-ADA is implemented (explained in the section "Image generation using StyleGAN2-ADA for dataset expansion"). This approach ensures that underrepresented classes have a sufficient number of samples for training, improving the model’s generalization and classification accuracy.

### Image generation using StyleGAN2-ADA for dataset expansion

One of the key challenges in training deep learning models for dental caries classification is the class imbalance in real-world datasets. In our dataset, Class III has significantly fewer samples compared to Class I and Class II. Such an imbalance can lead to biased model predictions where the classifier favours majority classes, poor generalisation, reduced accuracy for underrepresented caries types, and suboptimal feature learning, as deep learning models require sufficient data per class for effective training.

To ensure balanced class distribution and improved model generalization, we aim to expand each class into 600 total images by generating synthetic samples using StyleGAN2-ADA(Adaptive Discriminator Augmentation). This approach helps increase the training data diversity, reduce model overfitting, equalise the representation across Class I, II, and III, ensure unbiased learning, and improve the robustness of feature extraction by introducing high-quality synthetic images. StyleGAN2-ADA is an advanced generative adversarial network(GAN), capable of producing highly realistic synthetic images. It includes adaptive discriminator augmentation, ensuring stable training on limited real data by applying dynamic augmentations, high fidelity image synthesis generating detailed, high-resolution intraoral periapical radiographs with realistic textures, and class-conditional image generation allowing targeted augmentation of underrepresented classes (Table 2).

### Proposed framework

**Fig. 2:**
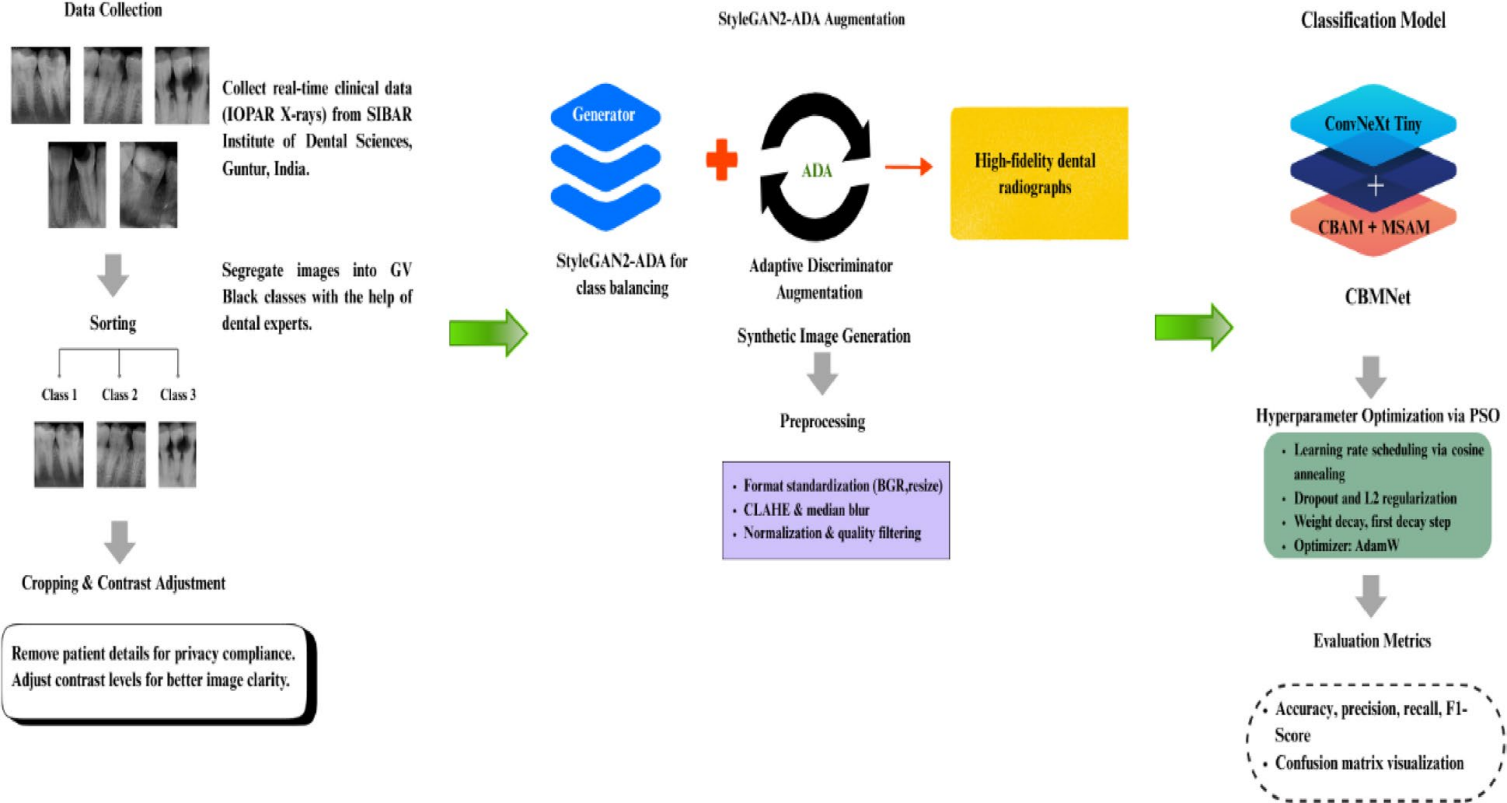
Overview of the proposed methodology for caries classification.

The proposed method groups dental caries into three types using intraoral periapical radiographs and advanced image enhancement, attention-augmented convolutional feature extraction, and a new training strategy. The design uses the lightweight and expressive ConvNeXt-Tiny backbone, which is improved by CBAM and a new Multi-Scale Attention Module (MSAM) to make it easier to find diagnostic information. Particle Swarm Optimization (PSO) finds the best hyperparameters, and cross-validation and test-time augmentation (TTA) are used to check the model to make sure it can generalize well. The next sections go into more detail on the main parts of this framework (Fig. 2).

#### Backbone architecture: ConvNeXt-Tiny

ConvNeXt-Tiny is the most important part of our framework for gaining features. Liu et al.^33^ built ConvNeXt, a convolutional neural network (CNN) that combines ResNet-style structures with ideas from Vision Transformers. Instead of Batch Normalization, this method uses GELU activation functions, bigger convolutional kernels (like 7 × 7), and Layer Normalization. The smallest form of the family is ConvNeXt-Tiny. It strikes a good mix between speed and accuracy, which makes it great for looking at medical images where speed is quite crucial, especially in real-time clinical situations. The picture you put in is $\:\varvec{X}\in\:{\mathbb{R}}^{H\times\:W\times\:3}$. To achieve the feature representation in Eq. (1), you need to travel through a lot of ConvNeXt blocks:

1 $$\:{\:F=}\phi_{ConvNeXt}\left(\varvec{X}\right)\in\:{\mathbb{R}}^{h\times\:w\times\:C}$$

where $\:{\:}\phi_{ConvNeXt}$denotes the sequence of depthwise convolutional operations and *C* is the number of output channels.

Our analysis employs the pretrained ConvNeXt-Tiny model without the top classification head. Instead, we create a customized attention-based classification head on top of the backbone. This allows the model to benefit from general visual features acquired from ImageNet while still being fine-tuned to recognize tiny patterns in greyscale dental radiographs. The model input is downsized to 224 × 224 in 3-channel BGR format for compliance with the pretrained backbone.

#### Attention modules: CBAM and MSAM

**Fig. 3:**
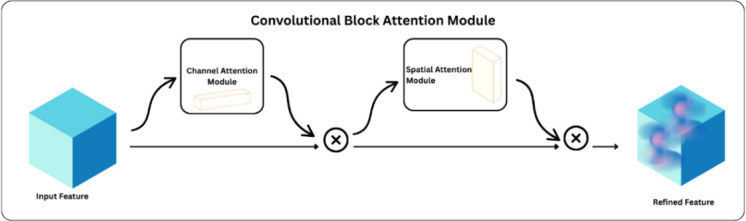
Architecture of Convolutional Block Attention Module.

**Fig. 4:**
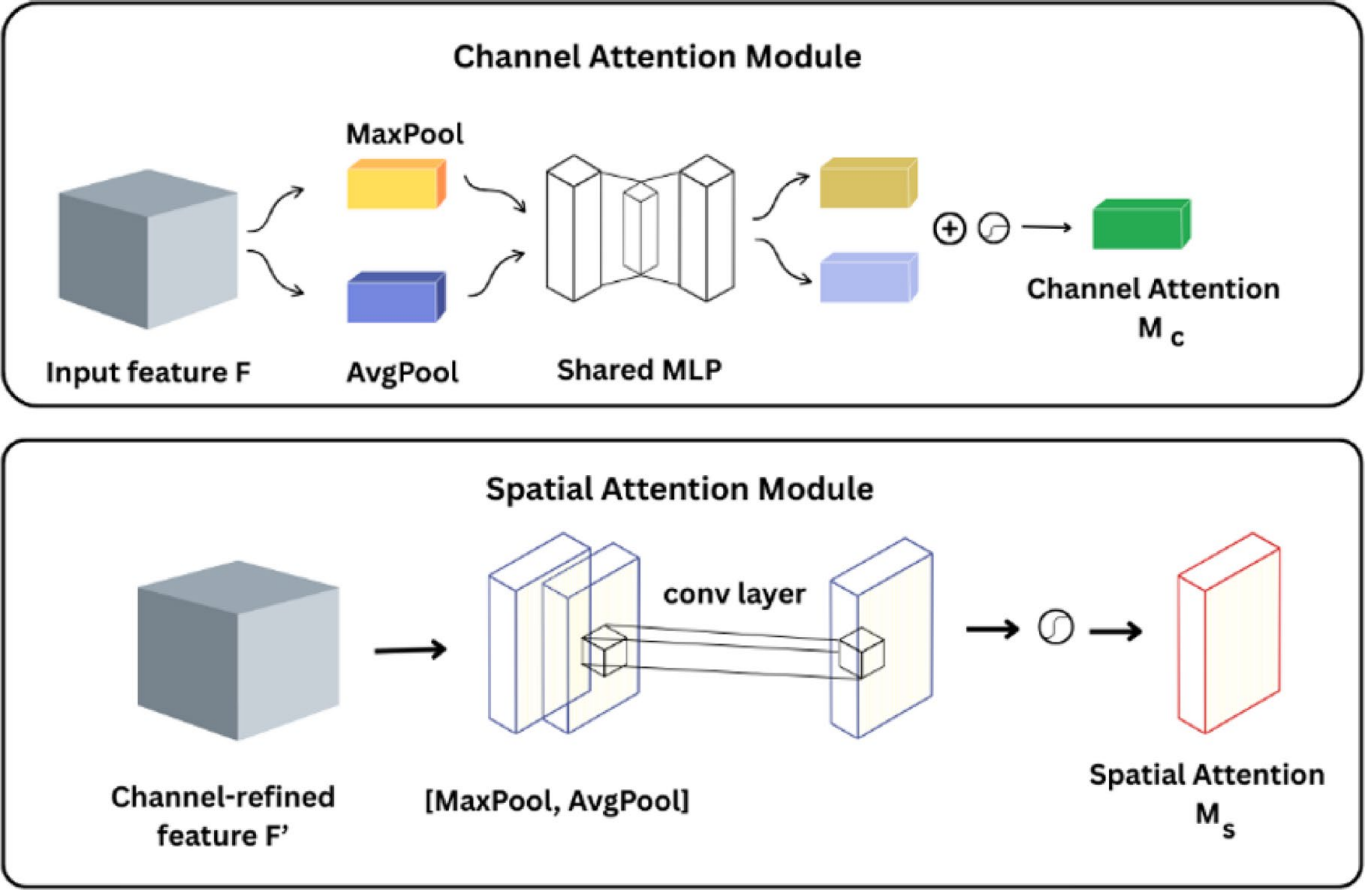
Channel and Spatial Attention Modules of CBAM.

We use the Convolutional Block Attention Module (CBAM) and the Multi-Scale Attention Module (MSAM) to help the model pay greater attention to things that are relevant in a clinical setting. Figure 3 shows that CBAM^34^ is a common module that uses attention in both the channel and spatial dimensions. The channel attention method uses global average and max pooling to produce a descriptor for each feature map. This makes the network focus more on the channels that are most important. Then, by pooling along the channel axis, spatial attention is used to make a spatial map that shows the most important places in each feature map. When used together, these enable the model get rid of the background noise and show lesions or areas of damage in the radiograph. Figure 4 shows that CBAM uses spatial and sequential channel attention on a feature map F. As illustrated in Eq. (2), the channel attention module employs the global average pooling (GAP) and max pooling (GMP) methods to find a descriptor.

2 $$\:{M}_{c}=\sigma\:\left(MLP\left(GAP\left(F\right)\right)+MLP\left(GMP\left(F\right)\right)\right)$$

where σ denotes the sigmoid activation and MLP(∙) is a shared multi-layer perceptron. The attention-weighted feature is shown in Eq. (3):

3 $$\:{F}^{{\prime\:}}={M}_{c}\odot\:F$$

Next, spatial attention computes :

4 $$\:{M}_{s}=\sigma\:\left({f}^{7\times\:7}\left(\left[AvgPool\left({F}^{{\prime\:}}\right);MaxPool\left({F}^{{\prime\:}}\right)\right]\right)\right)$$

where $\:{f}^{7\times\:7}$ is a convolutional layer with kernel size 7 × 7 and the output is shown in Eq. (5) :

5 $$\:{F}^{{\prime\:}{\prime\:}}={M}_{s}\odot\:{F}^{{\prime\:}}$$

**Fig. 5:**
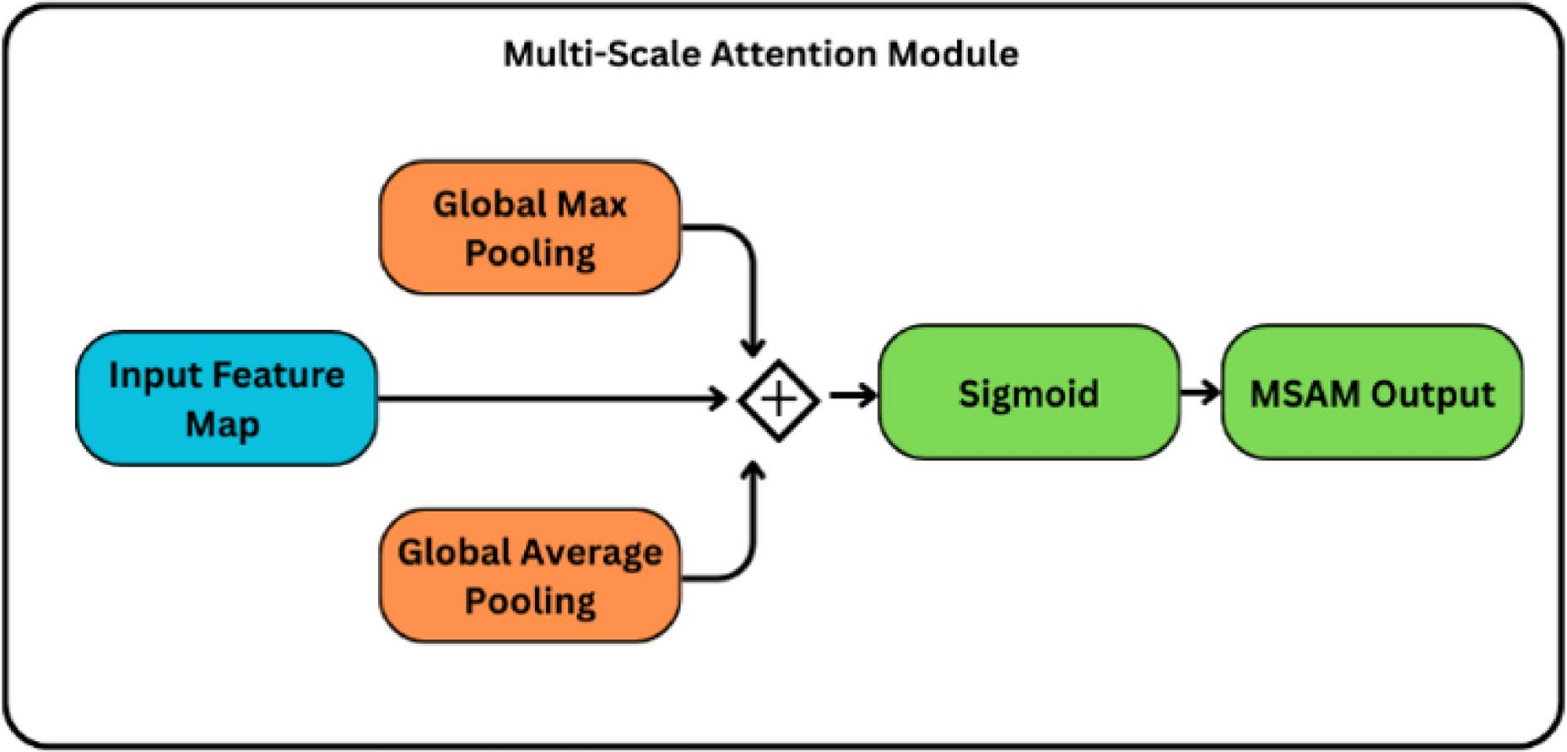
Multi-scale attention module.

In addition, we offer a Multi-Scale Attention Module (MSAM), which is specifically created to improve feature representation. The input to MSAM is the refined feature map $\:{F}^{{\prime\:}{\prime\:}}\in\:{\mathbb{R}}^{h\times\:w\times\:C}$, typically [7 × 7 × 768] after CBAM. This module computes global average and maximum pooled features, merges them, and uses a 1 × 1 convolution to generate attention weights across scales as shown in Fig. 5. MSAM enables the model to record features of changing granularity, which is critical in dental imaging because lesions might present as modest changes in texture, size, or contrast. Let $\:{g}_{avg},{g}_{max}\in\:\:{\mathbb{R}}^{C}\:$ be global descriptors obtained by applying global average pooling (GAP) and global max pooling(GMP) over the spatial dimensions of $\:{F}^{{\prime\:}{\prime\:}}$. These vectors are summed element-wise and reshaped to $\:{\mathbb{R}}^{1\times\:1\times\:C}$ to match the spatial format required for channel-wise broadcasting when applied to the input feature tensor. MSAM computes:

6 $$\:{M}_{ms}=\sigma\:\left({Conv}_{1\times1}\left(Reshape\left({g}_{avg}+{g}_{max}\right)\right)\right)$$

and the resulting $\:{M}_{ms}\in\:\:{\mathbb{R}}^{1\times\:1\times\:C}\:$is then broadcast-multiplied with $\:{F}^{{\prime\:}{\prime\:}}$ to obtain the final attended tensor $\:{F}^{*}$as shown in Eq. (7):

7 $$\:{F}^{*}={M}_{ms}\odot\:{F}^{{\prime\:}{\prime\:}}$$

This combination improves attention across both local features and global structure variations common in carious lesions. Just as a dentist may first step back to view an entire radiograph for overall context and then zoom in with focused attention on a suspicious region, CBAM and MSAM help the model shift between broad and fine perspectives. CBAM ensures the model identifies what and where to focus, while MSAM ensures it considers both small cavities and larger lesion patterns across scales. Together, these dual-attention modules enhance the network’s ability to mimic a clinician’s systematic approach to radiographic examination, thereby reducing the risk of missing early-stage or subtle lesions.

#### Data augmentation and preprocessing

To improve generalization during model training, we apply on-the-fly data augmentation using the *albumentations* library. This is distinct from StyleGAN2-ADA, which we use earlier to augment the dataset with synthetic samples. Let $\:A\left(\bullet\:\right)$ denote the augmentation function:

8 $$\:\stackrel{\sim}{X}=A\left(X\right)$$

Transformations include:

- Random flips $$\left( {x,y} \right) \in \left\{ {0,1} \right\}$$.
- Affine transforms with scale $\:s \sim U\left( {{\text{0}}{\text{.9,1}}{\text{.1}}} \right)$.
- Brightness and contrast adjustments.
- Gaussian, motion, and median blur.
- Elastic deformations.

The final input $\widetilde{{X~}} \in {\mathbb{R}}^{{224 \times 224 \times 3}}$ is normalized using the pretrained ConvNeXt preprocessing pipeline.

#### Hyperparameter optimization via PSO

We employ Particle Swarm Optimization (PSO)^35^ to search the space of training hyperparameters in order to prevent manual hyperparameter adjustment and guarantee ideal training circumstances. The population-based optimization algorithm PSO, which draws inspiration from nature, mimics the motion of particles (solutions) in a search space and updates them according to both global and personal best locations. This method effectively searches the space without the need for gradient information.

Let the hyperparameter vector for a given solution (or particle) be defined as:

9 $$\:{x}_{i}=\left[\eta\:,\omega\:,{d}_{1},{d}_{2},{f}_{ds},\lambda\:\right]$$

where:

- $\:\eta\:$ is the initial learning rate.
- $\:\omega\:$ is the weight decay.
- $\:{d}_{1},{d}_{2}$ are dropout rates in the classification head.
- $\:{f}_{ds}$ is the first decay step in cosine annealing.
- $\:\lambda\:$ is the L2 regularization strength.

Each particle’s velocity and position are updated per iteration $\:t$ using Eqs. (10) and (11):

10 $$\:{v}_{i}^{\left(t+1\right)}=w.{v}_{i}^{\left(t\right)}+{c}_{1}{r}_{1}\left({p}_{i}^{best}-{x}_{i}^{\left(t\right)}\right)+{c}_{2}{r}_{2}\left({g}^{best}-{x}_{i}^{\left(t\right)}\right)$$

11 $$\:{x}_{i}^{\left(t+1\right)}={x}_{i}^{\left(t\right)}+{v}_{i}^{\left(t+1\right)}$$

where:

- $\:w$ is the inertia weight.
- $\:{c}_{1},$ $\:{c}_{2}$ are acceleration coefficients.
- $r_{1} ,~r_{2} \sim ~U\left( {0,1} \right)$ are random samples.
- $\:{p}_{i}^{best}$ is the personal best solution of particle $\:i$.
- $\:{g}^{best}$ is the global best across the swarm.

Just as multiple dentists might examine the same radiograph and share insights before reading a final consensus, PSO enables multiple “agents” to explore the solution space and converge on the most reliable set of hyperparameters. This process reduces the risk of the model “overfitting” to suboptimal parameter choices, thereby ensuring more stable classification performance.

Fitness Function Design.

The objective function minimized by PSO is shown in Eq. (12):

12 $$\:{\mathcal{L}}_{PSO}\left({x}_{i}\right)=1-\underset{e}{\text{max}}({ValAccuracy}^{\left(e\right)}\left({x}_{i}\right))$$

where $\:{ValAccuracy}^{\left(e\right)}$ is the validation accuracy at epoch $\:e$ when training the model with the hyperparameters $\:{x}_{i}.$ The inverse formulation ensures that higher validation accuracy leads to lower cost, guiding the swarm towards more performant configurations. To avoid data leakage, we isolate a portion of the training data into a dedicated train-validation split for the optimization process. The test set is never used during tuning. Once the optimal hyperparameters are obtained, we retrain the model on the full training set and evaluate it on the held-out test set to ensure an unbiased assessment. Each model is trained for 10 epochs with early stopping (patience = 3) to reduce the computational burden. The fitness function tracks and updates the best validation accuracy across epochs.

#### Classification head and loss function

The classification head consists of:

- GlobalAveragePooling layer.
- Dense layer with 256 units and ReLU activation.
- Two dropout layers with PSO-optimized dropout rates.
- Final SoftMax output layer for 3-class classification.

We use focal loss^36^ instead of traditional categorical cross-entropy to lessen the impact of easy samples controlling the gradient. Let $\:{p}_{t}=softmax\left({y}_{true},{y}_{pred}\right),$ the focal loss is defined as in Eq. (13):

13 $$\:{\mathcal{L}}_{focal}=-{\alpha\:}_{t}{\left(1-{p}_{t}\right)}^{\gamma\:}\text{l}\text{o}\text{g}\left({p}_{t}\right)$$

where $\:\gamma\:=1.5\:and\:\alpha\:=0.25.\:$This loss penalizes easy examples and focuses the training on harder misclassified ones, particularly useful when class distributions are uneven.

#### Test-time augmentation (TTA)

During final inference, we employ Test-Time Augmentation (TTA) to increase the prediction’s robustness. Every test image in TTA undergoes a number of stochastic augmentations, such as flipping and contrast alteration, several times. The final classification score is then calculated by averaging the predictions from these modified versions. This process can be likened to how a dentist might tilt a radiograph under different light angles or zoom levels to ensure that subtle carious lesions are not missed. Each “view” provides slightly different information, and the dentist synthesizes these impressions before making a diagnosis. Similarly, TTA stabilizes CBMNet’s predictions by testing the model across multiple variations of the same radiograph, reducing the chance of misclassifying faint or borderline lesions.

On the test set, we used TTA with five augmentation rounds. Let $\:{\left\{{A}_{i}\right\}}_{i=1}^{K}$ be a set of augmentations. For each test image $\:X$, we compute:

14 $$\:\widehat{y}=\frac{1}{K}\sum\:_{i=1}^{K}f({A}_{i}\left(X\right))$$

where $\:f(\bullet\:)$ is the trained model and $\:\widehat{y}$ is the final predicted class probability vector. TTA mitigates prediction variance due to input noise and boosts reliability in real-world settings.

#### Sample size justification (power analysis)

To justify the adequacy of our evaluation set, we performed a one-sample superiority power analysis on the primary endpoint of overall accuracy. Using a conservative benchmark accuracy of $\:{p}_{0}=0.85,$ a two-sided significance level of $\:\alpha\:=0.05,$ and target power of 80%, the required test size is $\:n\approx\:176$ if the true accuracy is $\:{p}_{1}=0.92,$ and $\:n\approx\:363$ if $\:{p}_{1}=0.90.$ Our held-out test set contained *n*=360 radiographs (120 per class), which provides ≥ 80% power for accuracies of 0.90 or greater and > 98% achieved power for the observed accuracy of 0.92. This analysis confirms that the selected sample size was statistically sufficient to support the reliability of our reported findings.

Figure 2 illustrates the overall workflow of the proposed framework, encompassing synthetic image generation, preprocessing pipeline, classifier training, and evaluation. This end-to-end architecture integrates data augmentation using StyleGAN2-ADA, quality-controlled preprocessing, and a ConvNeXt + CBAM + MSAM classifier to achieve robust and interpretable dental caries classification.

## Experimental configuration

This section talks about how the proposed CBMNet studied for partial G.V. Black classification on intraoral periapical radiographs. We elaborate on the techniques we employed to prepare the data, train the model, configure the classifier, and evaluate performance.

### Preprocessing

The preparation pipeline is specifically designed to handle three major issues that arise when classifying clinical radiographs: variances in image quality, an unequal dataset, and generalization during training. We carefully address each of these issues by using a combination of BRISQUE (Blind/Referenceless Image Spatial Quality Evaluator) score evaluation, StyleGAN2-ADA-based synthetic data generation, and planned data augmentation.

#### Image quality assessment using BRISQUE

We use the BRISQUE metric to check the quality of the images to make sure that we can reliably extract features from intraoral periapical radiography. Mittal et al.^37^ came up with BRISQUE, a way to measure image quality without a reference image. It looks at natural scene statistics in the spatial domain to find perceptual distortions like blur, noise, and overexposure, which are prevalent problems in real-time clinical imaging. A lower BRISQUE score means higher visual quality. The scores usually range from 0 (perfect) to 100 (very bad). But in some situations, especially with photographs that have been heavily edited or made to look clean with software, the BRISQUE score may be less than zero. This happens because of how the trained regression model works in some cases (like PIQ), and it doesn’t mean there is an error. We found a handful of these negative scores in our dataset. They are still valid and just show that the visual quality is far better than the model’s statistical baseline.

For dental radiography, it is very important that images are clear so that fine features like enamel edges, carious lesions, and pulp boundaries may be seen. Changes in exposure or motion errors can hide these features, which can make the model work less well. Since BRISQUE picks up on these distortions, it was a good filter to get rid of images that weren’t good enough to help with classification.

**Fig. 6:**
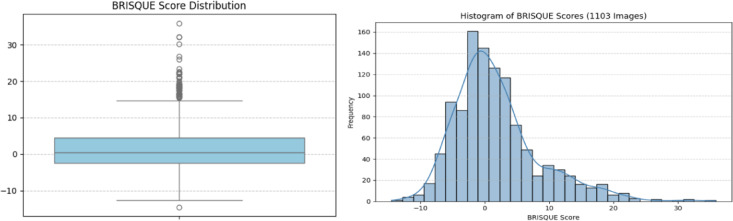
Distribution of BRISQUE scores across all 1103 collected dental radiographs.

We employ 1103 dental X-rays, all of which have BRISQUE scores of 40 or less. We chose this threshold based on what we could see and how dental X-rays are made. They usually have higher BRISQUE values than natural photos since they have a grayscale texture, low contrast, and sparse structure. A lot of research suggests that natural images should have thresholds between 20 and 30, however we discover that a cutoff of 40 keeps relevant diagnostic data while also keeping quality control. We use both a histogram and a boxplot (see Fig. 6) to show the distribution of BRISQUE scores across every image in order to explain why we chose this one. These graphs indicate that most of the scores are significantly below the 40 marks, and some high-quality photographs even have negative values. Table 3 also has a descriptive overview of the BRISQUE scores, which shows that the average score was 1.8075, the standard deviation was 6.6510, and the lowest score was − 14.4583. This backs up what we have seen in real life: medical images can be different from natural photos, which is why we have a relaxed yet selective threshold.

**Table 2:** StyleGAN2-ADA training Parameters.

Parameter	Value/Setting
Model architecture	StyleGAN2-ADA (initialized from pre-trained FFHQ-512 model)
Hardware/Environment	Google Colab Pro, NVIDIA A100 GPU, Python 3.9.21
Libraries	torch = = 1.1.1 + cu110, torchvision = = 0.8.2 + cu110, numpy = = 1.23.5
Input Size	512 × 512
Batch Size	32
Augmentation	Adaptive Discriminator Augmentation
Training Length	kimg = 2500
Learning rate (initial)	G_lr = 0.0025, D_lr = 0.0025
Learning rate (fine-tuned)	G_lr = 0.0004, D_lr = 0.0004
R1 regularization weight	γ = 6.55 → 8 (fine-tuned)
EMA (exponential moving avg)	2.5 → 14 (fine-tuned)

This filtering process standardizes the perceptual fidelity of both real and synthetic images, supports stable GAN training, and improves downstream classification accuracy.

#### Synthetic image generation using StyleGAN2-ADA

To address the imbalance in our dataset we employ StyleGAN2-ADA^38^, a generative adversarial network architecture specifically designed for high-fidelity image synthesis under limited data conditions. The key parameter settings are summarized in Table 2. This model integrates adaptive discriminator augmentation (ADA), which dynamically adjusts image-level augmentations during training to stabilize GAN performance in small datasets. We implement the model on Google Colab Pro using an NVIDIA A100 GPU, running Python 3.9.21 and compatible deep learning libraries including $\:\text{t}\text{o}\text{r}\text{c}\text{h}==1.1.1+\text{c}\text{u}110,\:\text{t}\text{o}\text{r}\text{c}\text{h}\text{v}\text{i}\text{s}\text{i}\text{o}\text{n}==0.8.2+\text{c}\text{u}110$, and $\:\text{n}\text{u}\text{m}\text{p}\text{y}==\text{1.23.5}.\:$ All grayscale radiographs are converted to 3-channel RGB format, resized to 512 × 512 pixels, and saved in uint8 format as required by the StyleGAN2 pipeline. Augmentations such as random rotations(± 10°, *p* = 0.8) and horizontal flips (*p* = 0.5) are applied specifically to minority classes, ensuring intra-class diversity. A target size of 490 images per class is achieved post-filtering.

**Table 3:** Descriptive summary of BRISQUE scores for 1103 dental Radiographs.

Statistic	Value
Count	1103
Mean	1.8075
Std Dev	6.6510
Min	−14.4583
25%	−2.4280
Median	0.5129
75%	4.4553
Max	35.8327

**Fig. 7:**
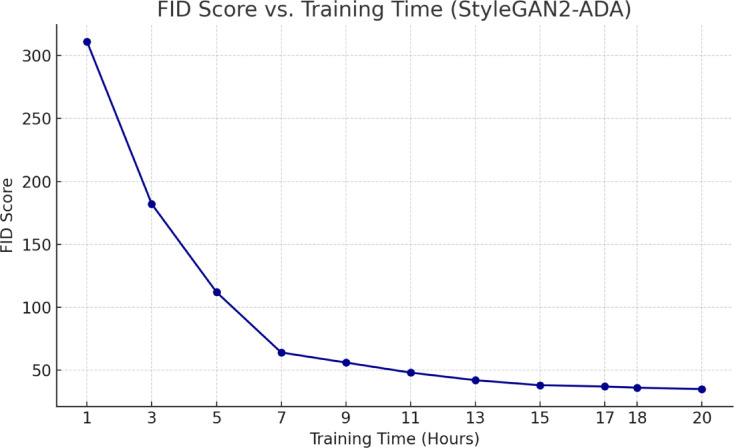
FID Trend over training time.

Training is performed using the following settings: $\:\text{batch}=32$, $\:\text{mirror}=1$, $\:\text{aug}=\text{ada}$, $\:\text{cfg}=\text{auto}$, $\:\text{metrics}=\text{fid}50\text{k}\_\text{full}$, $\:\text{snap}=10$, $\:\text{kimg}=2500$, and initialized from a pre-trained FFHQ-512 model. Progressive training snapshots are evaluated using Fréchet Inception Distance(FID) - a standard metric that quantifies the similarity between real and generated image distributions. Lower FID scores indicate more realistic outputs. Our training begin with a high FID of 311, and through staged optimization – first with $\:{G}_{lr}=0.0025,\:{D}_{lr}=0.0025,\:$gamma=6.55, ema=2.5, and later fine-tuned down to $\:{G}_{lr}=0.0004,\:{D}_{lr}=0.0004,$ gamma=8, ema=14 we have achieved a final FID score of 35, which falls within an acceptable range (typically, FID<50 is considered excellent for clinical-style images). The FID trend over the training time is shown in Fig. 7.

**Fig. 8:**
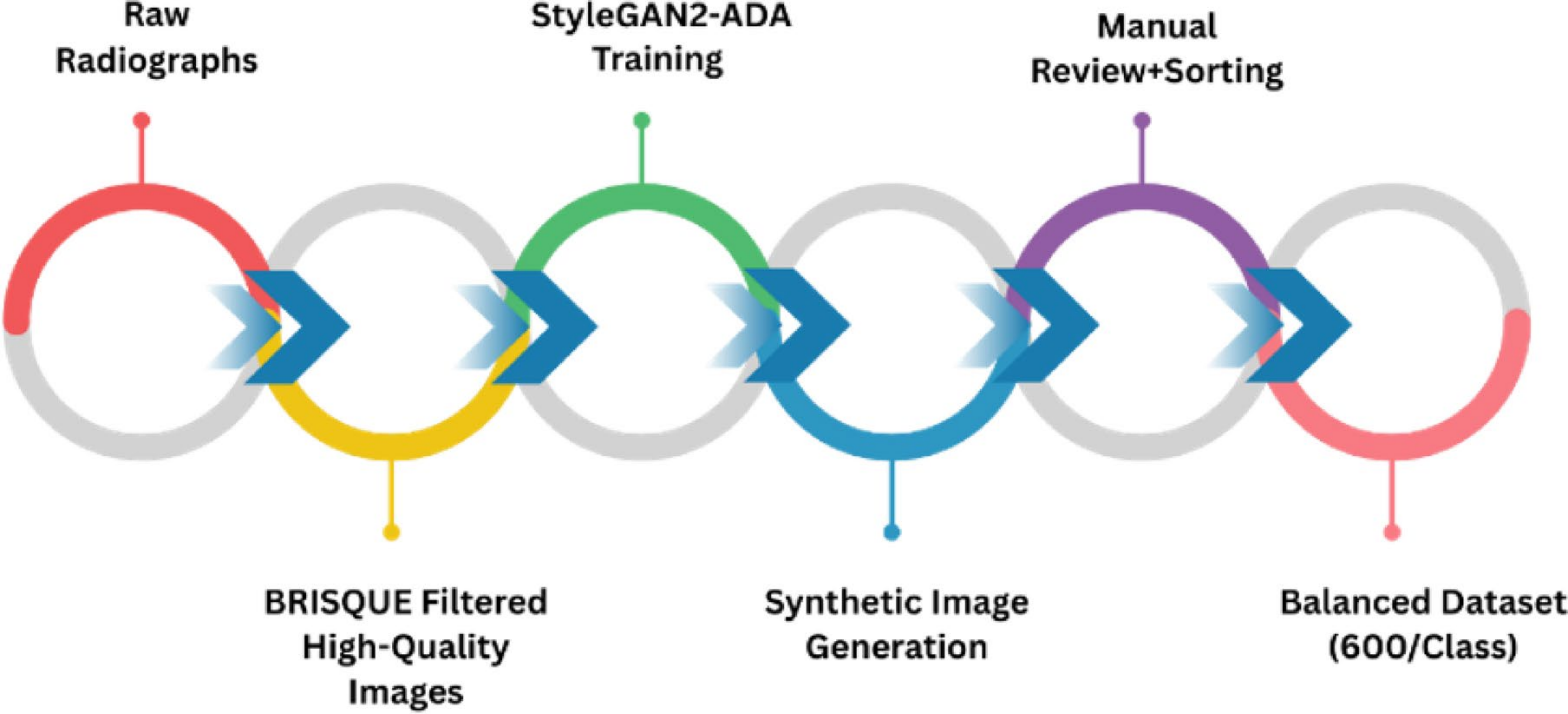
Pipeline for synthetic data preparation using StyleGAN2-ADA.

**Fig. 9:**
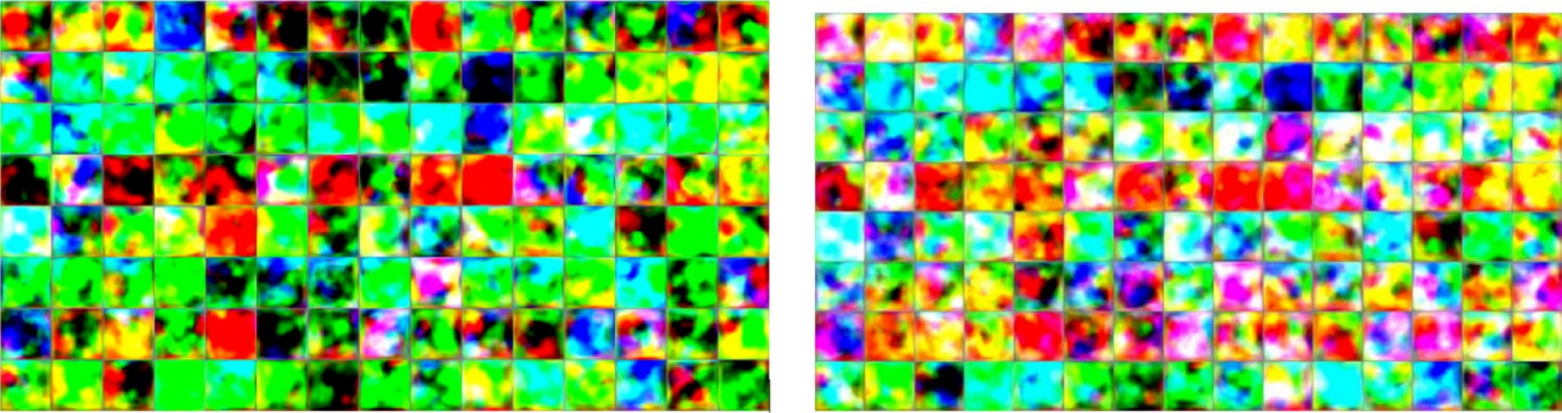
Initial Generated Images.

**Fig. 10:**
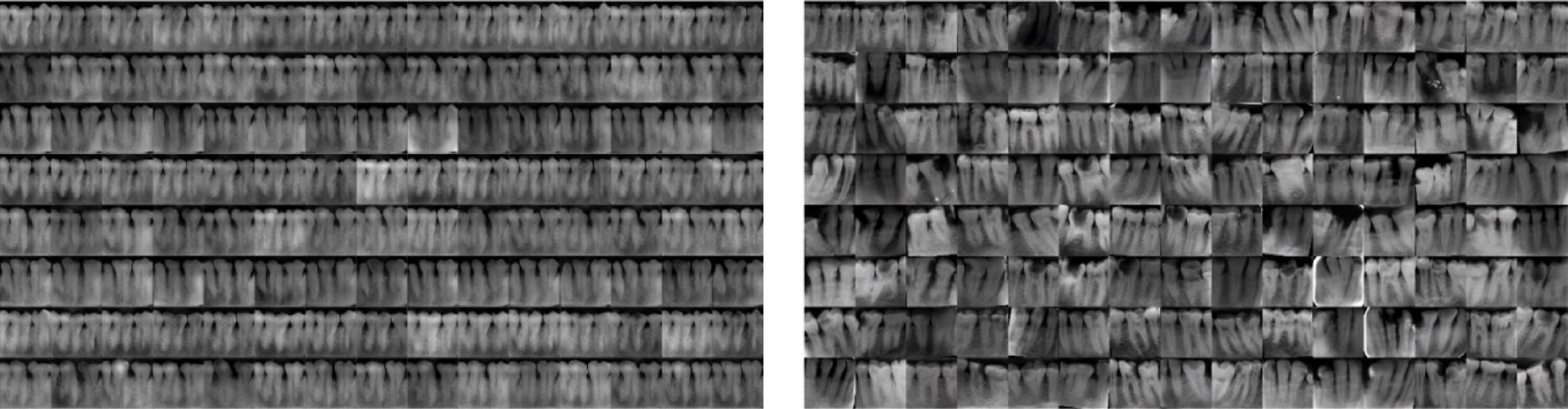
Generated Images During Training.

**Fig. 11:**
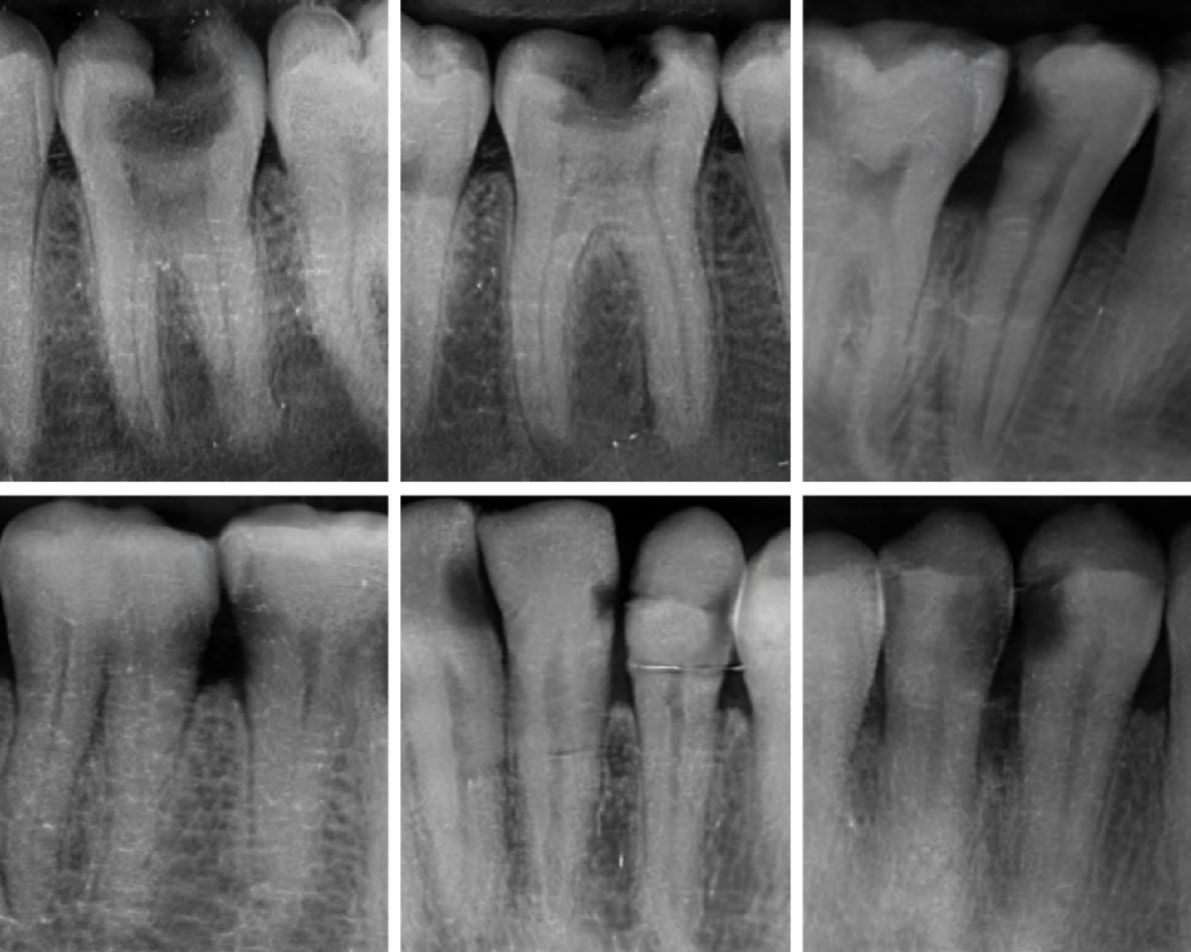
Representative synthetic intraoral periapical radiographs generated using the StyleGAN2-ADA frame work.

The process leading to a class-balanced dataset through StyleGAN2-ADA training and manual validation is summarized in Fig. 8. After training, synthetic samples are generated from the final best-performing snapshot, visually inspected, and sorted manually into respective G.V. Black classes. The synthetic images created at different phases of training are shown in Figs. 9 and 10. Vague or structurally distorted outputs are discarded. Sample synthetic radiographs generated using the trained StyleGAN2-ADA model are presented in Fig. 11. These images closely resemble real intraoral periapical X-rays and are visually inspected for anatomical plausibility and quality. This synthetic augmentation substantially improves dataset balance, visual realism, and class coverage.

To maintain clinical authenticity, we ensure that real images always outnumber synthetic ones across all classes. Specifically, for the most underrepresented class (Class III), we perform targeted minor augmentations(rotation, flip, brightness) on the 205 original images, generating 248 additional augmented real samples and raising the total real image count to 453. Following this, synthetic images generated via StyleGAN2-ADA are added to achieve a final balanced dataset of 600 images per class. The composition as shown in Table 4 is as follows: Class I – 408 real, 192 synthetic; Class II – 490 real, 110 synthetic; and Class III – 453 real, 147 synthetic. This carefully curated dataset ensures adequate class balance while preserving the integrity of real clinical data for robust and fair model training.

**Table 4:** Class-wise distribution of real and synthetic images.

	Class I	Class II	Class III
Real	408	490	453
Synthetic	192	110	147
Total	600	600	600

#### Human evaluation via blinded review protocol

To evaluate the perceptual realism of the synthetic radiographs generated by our StyleGAN-based model, we conducted a blinded human review involving domain experts. This evaluation determines whether dental professionals can distinguish real and synthetic radiographs based solely on visual cues.

**Fig. 12:**
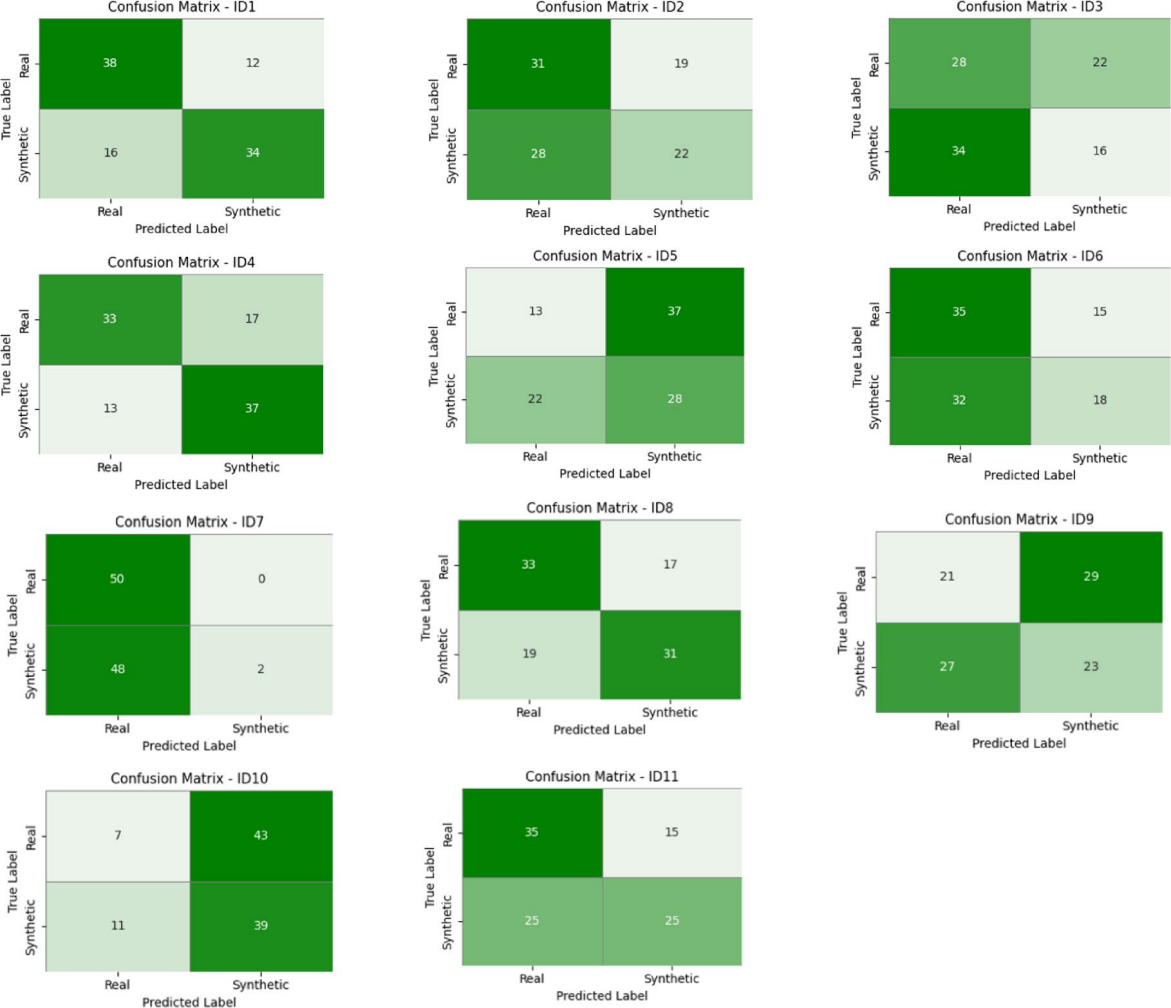
Confusion matrices of 11 dental experts (ID1–ID11) from the blinded study evaluating real vs. synthetic radiographs.

We gave each of the 11 reviewers a Google form with 100 randomly shuffled intraoral periapical radiographs. Half of the images were real and half were GAN-generated. We made sure that the reviewers didn’t know where the photographs came from or how many of them were real or fake. We asked them to label each image as either “Real” or “Synthetic” and give it a confidence score if they wanted to show their surety of their choice. We put together the response sheets and compare the predictions of the reviewers with the real results to get individual accuracy scores. The statistics showed that reviewers get an average score of 54.45%, with values ranging from 41% to 72%. The results conveyed that our synthetic images often look a lot like real radiographs, which makes it hard to tell the difference, even for educated dental specialists. This human review gave us significant proof that our generated images seem natural and could be used in education, augmentation, and diagnostic training procedures. Table 5 shows the accuracy of the classification by reviewer. Figure 12 shows the confusion matrices for all 11 dental specialists who took part in the blind evaluation. Some reviewers, like ID9 and ID10, showed almost random classification behaviour, while others, like ID7, showed very confident but uneven judgments, categorizing almost all images as real. Many specialists mistakenly thought that a lot of synthetic images were real, which showed that the generated images look a lot like real dental radiographs. This visual proof adds to the qualitative realism of the synthetic samples and shows how hard it is for people to tell the difference when they cannot see.

**Table 5:** Reviewer-wise classification accuracy in the blinded evaluation of real vs. Synthetic Images.

Reviewer ID	Accuracy(%)
ID1	72
ID2	53
ID3	44
ID4	70
ID5	41
ID6	53
ID7	52
ID8	64
ID9	44
ID10	46
ID11	60
	**Mean Accuracy : 54.45**

#### Standardized preprocessing for model input

To ensure uniformity and enhance the quality of the input data, all images – both synthetic and real – has undergone a consistent preprocessing pipeline. The primary objectives are to suppress noise and artifacts, enhance contrast, and standardize image dimensions and formats for optimal input to the classification network.

Initially, all images are loaded using OpenCV with support for various channel formats. Grayscale images are converted to 3-channel BGR, while RGBA images are stripped of the alpha channel. This conversion ensures that all inputs have a consistent 3-channel format, as required by the model. Each image is then resized to a fixed resolution of 224 × 224 pixels, maintaining consistency with the input dimensions of standard pretrained CNN architectures. To enhance visual contrast and highlight subtle dental features, Contrast Limited Adaptive Histogram Equalization (CLAHE) is applied to the grayscale version of each image. The equalized image is subsequently converted back to 3-channel BGR format. In the next step, a median blur filter with a kernel size of 3 is applied to reduce noise and suppress artifacts, which is particularly useful in radiographic images affected by acquisition inconsistencies or compression artifacts. Following enhancement and denoising, the pixel intensities are normalized to the range [0,1] by scaling with a factor of 1/255. The final pre-processed images are saved in PNG format for both training and testing datasets. The synthetic images are introduced only into the training set, and the test set comprises only real, original images. This separation ensures that no synthetic or augmented data leaks into the evaluation process, thereby preserving the validity of our reported performance metrics. The breakdown of real and synthetic images used in each set is provided in Table 6. Additionally, to assess the visual fidelity and eliminate low-quality images, we computed BRISQUE scores during an internal quality check phase. Images with poor perceptual quality are optionally filtered out before model training.

**Table 6:** Train-Test image Distribution.

	Train		Test (only real)
Class Type	Real	Synthetic	
Class 1	288	192	120
Class 2	370	110	120
Class 3	333	147	120
Total	1440		360

### Classifier

The proposed classifier CBMNet is based on the ConvNeXt-Tiny architecture, a modern convolutional neural network(CNN) design that maintains the efficiency of ResNet-style architectures while incorporating design principles from vision transformers. The model is initialized with pretrained ImageNet weights and adapted to accept input images of resolution 224 × 224 × 3. The $\:includ{e}_{top}=False\:$parameter removes the default classification head, allowing for integration with attention mechanisms and task-specific dense layers.

The ConvNeXt backbone outputs a final feature map of shape [B, H, W, C]=[B, 7, 7, 768], where B is the batch size and C = 768 is the number of channels. The Convolutional Block Attention Module (CBAM) and the Multi-Scale Attention Module (MSAM) are the two attention modules that this high-level representation is successively passed through. In the context of clinical imaging, where discriminative patterns may be subtle and locally confined, these attention blocks are especially useful because they concentrate the model’s capacity on the most pertinent spatial and channel-wise features.

In the CBAM, channel attention is first computed from the ConvNeXt feature map $\:\varvec{X}\in\:{\mathbb{R}}^{7\times\:7\times\:768}$. This is done by applying global average pooling(GAP) and global max pooling (GMP) over the spatial dimensions, producing two descriptors: $\:gavg\in\:{\mathbb{R}}^{C}$, and $\:gmax\in\:{\mathbb{R}}^{C}$ (i.e., both are 1D vectors of shape [C] = [768]). These pooled features are passed through a shared multilayer perceptron with a reduction ratio *r* = 8, consisting of $\:Dense(C/8,relu)$ followed by $\:Dense\left(C\right).\:$The outputs are summed and passed through a sigmoid activation to yield the channel attention map $\:{M}_{c}\in\:{\mathbb{R}}^{1\times\:1\times\:C}$, which is applied multiplicatively to the original feature map. Subsequently, spatial attention is computed by concatenating average and max pooled versions of the channel-attended tensor along the channel axis, followed by a $\:7\times\:7$ convolution and sigmoid activation. The resulting spatial attention map $\:{M}_{s}\in\:{\mathbb{R}}^{7\times\:7\times\:1}$ is again multiplicatively applied to enhance spatially salient regions.

To complement CBAM with global context modelling, the MSAM module computes a fused representation of GAP and GMP applied on the CBAM-refined feature map. The two pooled vectors, $\:gavg\:and\:gmax\in\:{\mathbb{R}}^{C},\:$are summed element-wise to form a global context descriptor, which is then reshaped to $\:{\mathbb{R}}^{1\times\:1\times\:C}$ to enable channel-wise broadcasted multiplication with the refined feature map. This reshaped vector goes via a 1 × 1 convolution and then a sigmoid activation, which makes the final attention map $\:{M}_{ms}\in\:{\mathbb{R}}^{1\times\:1\times\:C}.$ This map is also used to multiply the spatially attended feature tensor. These procedures are only multiplicative, which means that they highlight important features without adding any further noise.

The attended feature tensor is then globally average pooled and passed through a fully connected classification head. This head consists of:

- A dropout layer with rate $\:{d}_{1}:0.42$,
- A dense layer with 256 units and ReLU activation, regularized with L2 regularization ($\:\lambda\:):9.98e-4$.
- A second dropout layer with rate $\:{\:d}_{2}:0.16$,
- A final SoftMax output layer with three units corresponding to the target classes.

The entire model is optimized using the AdamW optimizer with a PSO-tuned initial learning rate of $\:\eta\:=3.93e-4$, weight decay $\:\omega\:=1.84e-5$, and a cosine decay restart schedule configured with first decay step $\:{T}_{0}=7,\:{t}_{mul}=2.0\:and\:{m}_{mul}=1.0.$ A batch size of 32 is used throughout the training process. Equation 13 explains the categorical focal loss, which is the objective function. This loss function is helpful for dealing with class imbalance and making sure that well-classified samples don’t cross over during training.

**Fig. 13:**
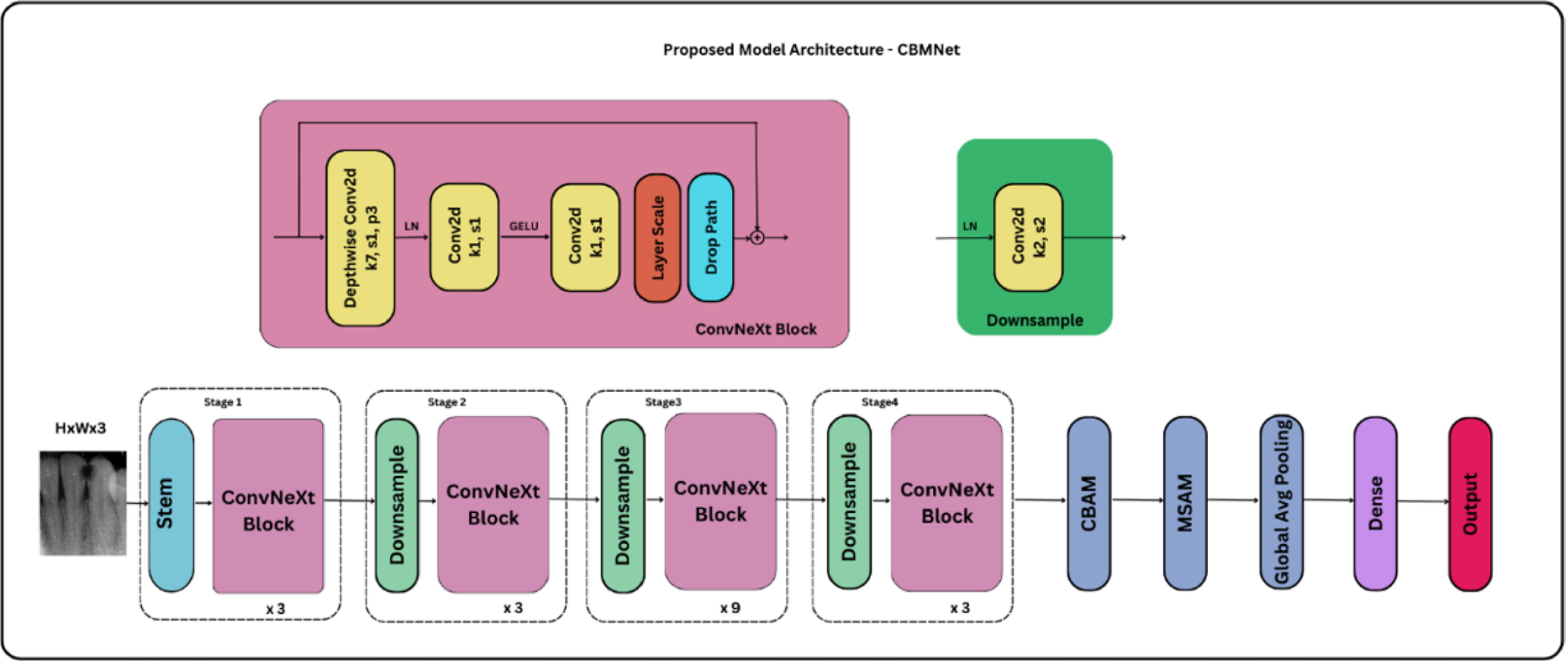
CBMNet Architecture.

The dual attention modules and well set hyperparameters help the model quickly discover and categorize the most important features in dental X-rays. This makes the classification function better, even when there isn’t a lot of data to work with. Figure 13 shows how the model is set up.

### Evaluation metrics

We use a set of standard measures for multi-class image classification tasks to fully evaluate how well the suggested classification model works. Not only do these measures show how accurate the model is overall, but they also show how well it can tell the difference between the different classes.

The primary evaluation metrics used in this study include:

- Accuracy: Accuracy is a way to see how well the model predicts across all classes. The ratio of correct forecasts to the total number of guesses is what it is.

15 $$\:Accuracy=\frac{TP+TN}{TP+TN+FP+FN}$$

16 $$\:Accuracy=\frac{1}{N}\sum\:_{i=1}^{N}1(\widehat{{y}_{i}}={y}_{i})$$

This is a generalized equation for multi-class:

where $\:\widehat{{y}_{i}}$ is the predicted label and $\:{y}_{i}\:$is the true label for instance $\:i,$ and $\:1(\bullet\:)$ 

is the indicator function. Accuracy is good for general performance, but it can be misleading when the data is not balanced, hence more measurements are needed.

- Precision (Per Class): Precision quantifies how many of the instances predicted as a given class are actually correct. For class c, precision is defined as in Eq. (17):

17 $$\:{Precision}_{c}=\frac{{TP}_{c}}{{TP}_{c}+{FP}_{c}}$$

High precision is critical in medical diagnostics where false positives could lead to unnecessary interventions or treatments.

- Recall(Per Class): Recall, or sensitivity, measures how many actual instances of class c were correctly identified:

18 $$\:{Recall}_{c}=\frac{{TP}_{c}}{{TP}_{c}+{FN}_{c}}$$

High recall is especially important in dental imaging, where missing a true caries case(false negative) could delay diagnosis and lead to complications.

- F1-Score(Per Class): The F1-score is the harmonic mean of precision and recall, which means it balances both:

19 $$\:{F1}_{c}=\frac{2.{Precision}_{c}.{Recall}_{c}}{{Precision}_{c}+{Recall}_{c}}$$

This metric is especially useful for classes that are not balanced, when optimizing for either precision or recall alone may not be enough.

- Macro Average: The macro average calculates the unweighted mean of a metric across all classes as shown in Eq. (20):

20 $$\:Macro-M=\frac{1}{C}\sum\:_{c=1}^{C}{M}_{c}$$

where $\:{M}_{c}$ is the value of the metric (precision, recall, or F1) for class c, and C is the number of classes. Macro averaging treats all classes equally, regardless of class size.

These measures are used to check how well the model works on the test dataset that was set aside. We used scikit-learn’s metric functions to calculate all of the reported values, which makes sure that the results are the same every time.

## Results

This section presents a comprehensive evaluation of the proposed CBMNet framework for multi-class classification of dental caries. The performance is assessed in three phases: hyperparameter optimization, cross-validation, and final testing (including test-time augmentation). Evaluation metrics are computed to quantify the model’s ability to generalize and maintain class-wise discrimination.

### Hyperparameter optimization results (PSO)

**Fig. 14:**
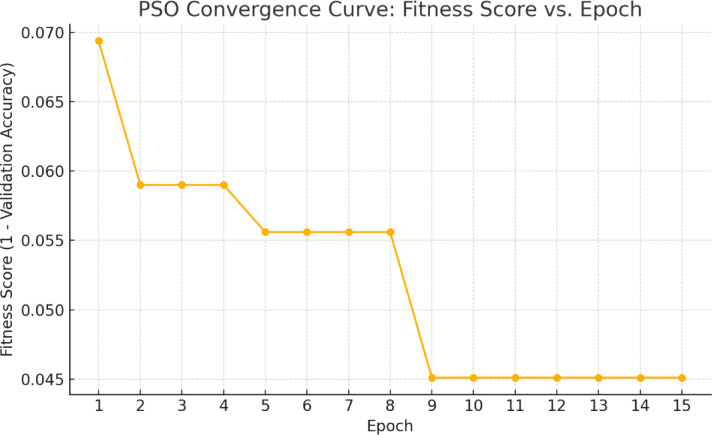
PSO convergence curve showing fitness improvement over epochs.

The search for optimal hyperparameters is conducted using Particle Swarm Optimization(PSO) over 15 iterations and a swarm size of 6. The goal is to minimize the validation loss and maximize classification performance on unseen data, as illustrated in Fig. 14.

The best fitness score attained is:

$$\:Current\:best:0.04514,\:\:Global\:best:0.04514$$

This corresponds to a validation accuracy of approximately 95.49% on the best-performing configuration. The hyperparameter vector achieving this score is:

- Initial learning rate $\:\left(\eta\:\right):3.93e-4$.
- Weight decay $$\:\left(\omega\:\right):1.84e-5$$.
- Dropout $$\:{d}_{1}:0.42,{\:d}_{2}:0.16$$.
- First decay step in cosine annealing$\:{\:(f}_{ds}):7$.
- L2 regularization ($$\:\lambda\:):9.98e-4$$.

These parameters are subsequently validated using 5-fold cross-validation.

### Cross-validation performance

**Fig. 15:**
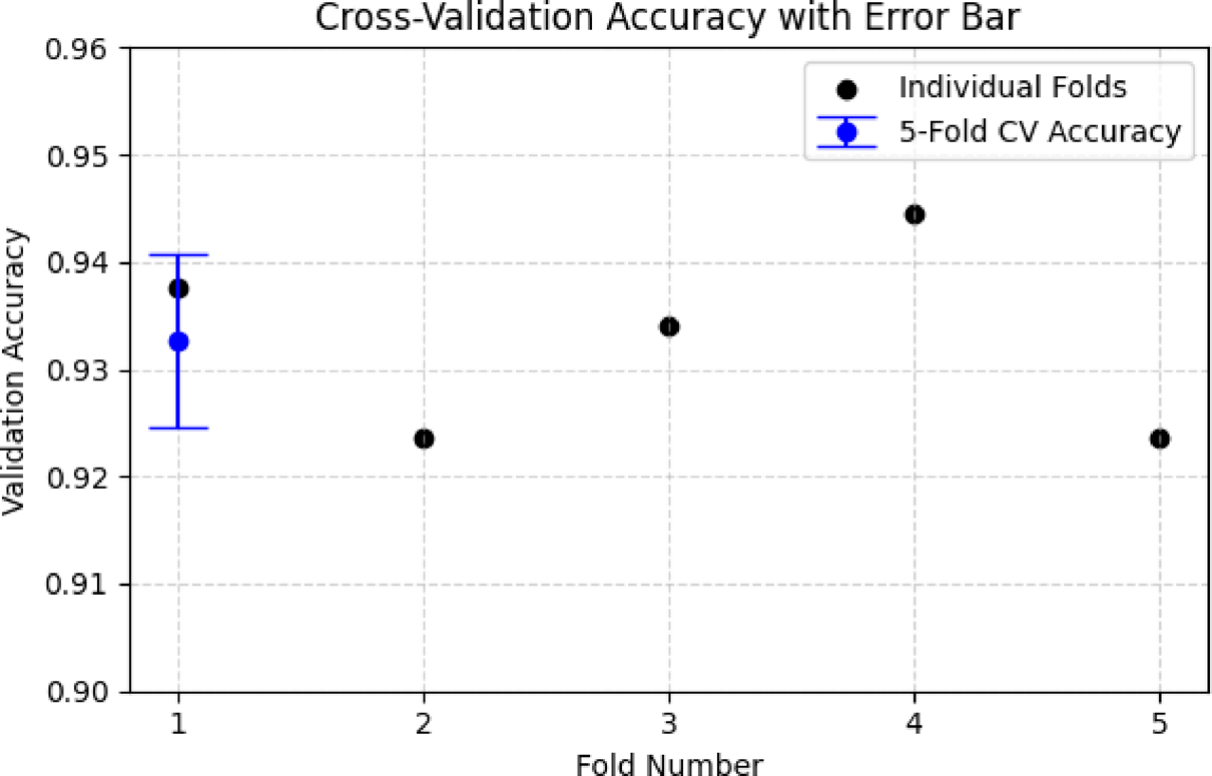
5-fold cross-validation accuracy with mean ± standard deviation.

To ensure the optimized hyperparameters’ robustness, stratified 5-fold cross-validation is performed. The dataset is split so that each fold preserves the original class distribution. The accuracy across folds, along with the mean and standard deviation, is presented in Fig. 15.

$$\:Fold-wise\:Validation\:Accuracies:[93.75\%,\:92.36\%,\:93.4\%,\:94.44\%,\:92.36\%]$$

The aggregated cross-validation performance yielded:

$$\:Mean\:Validation\:Accuracy:93.26\%,\:\:Standard\:Deviation:\pm\:0.81\%$$

This low variance across folds confirms that the selected hyperparameters are stable and generalize well across subsets of the data.

### Ablation studies

To rigorously evaluate the contribution of each component within the proposed CBMNet architecture, we perform a comprehensive ablation study. Specifically, we examine the impact of the Multi-Scale Attention Module (MSAM), Convolutional Block Attention Module (CBAM), and Test-Time Augmentation (TTA). We progressively introduce each component to establish their incremental contributions clearly. The models evaluated in this study include:

1. *Baseline:* ConvNeXt-Tiny without attention modules.
2. *Baseline + MSAM:* ConvNeXt-Tiny with the addition of only the MSAM attention block.
3. *Baseline + CBAM:* ConvNeXt-Tiny with the addition of only the CBAM attention block.
4. *Baseline + CBAM + MSAM:* ConvNeXt-Tiny with both CBAM and MSAM attention modules combined.
5. *CBMNet + TTA (proposed):* The final proposed model with both attention modules and the application of test-time augmentation during inference.

All models are trained and evaluated under identical hyperparameter settings obtained through Particle Swarm Optimization (PSO), ensuring that the observed performance variations are attributable solely to architectural differences rather than optimization discrepancies. For each ablation variant, training is performed using identical data splits and preprocessing steps as detailed in the experimental section "Classifier". All models use the same training parameters: batch size of 32, initial learning rate of $\:3.93e-4$, weight decay of $\:1.84e-5$, dropout rates of 0.42 and 0.16, focal loss, and cosine decay learning rate schedule.

Table 7 presents the performance metrics, including accuracy, precision, recall, and F1-Score, across all evaluated model configurations. We observe a clear incremental improvement in performance with the inclusion of each attention mechanism and TTA.

**Table 7:** Quantitative results of the ablation studies conducted on the held-out test set.

Model Configuration	Accuracy	Precision	Recall	F1-score
Baseline(ConvNeXt-Tiny	86.94	88.19	86.94	87.2
Baseline + MSAM	88.89	89.04	88.89	88.94
Baseline + CBAM	89.44	90.34	89.44	89.62
Baseline + CBAM + MSAM (CBMNet)	90.28	90.82	90.28	90.40
**CBMNet + TTA (proposed)**	**92**	**92**	**92**	**92**

Precision = Positive Predictive Value; Recall = Sensitivity; F1-score = Harmonic mean of precision and recall.

**Table 8:** Performance comparison of CBMNet with state-of-the-art deep learning models.

Model Architecture	Accuracy	Precision	Recall	F1-score
ResNet50	87.22	87.32	87.22	87.24
EfficientNetB0	89.16	89.81	89.16	89.29
DenseNet121	89.72	89.68	89.72	89.69
**CBMNet + TTA (proposed)**	**92**	**92**	**92**	**92**

Precision = Positive Predictive Value; Recall = Sensitivity; F1-score = Harmonic mean of precision and recall.

**Fig. 16:**
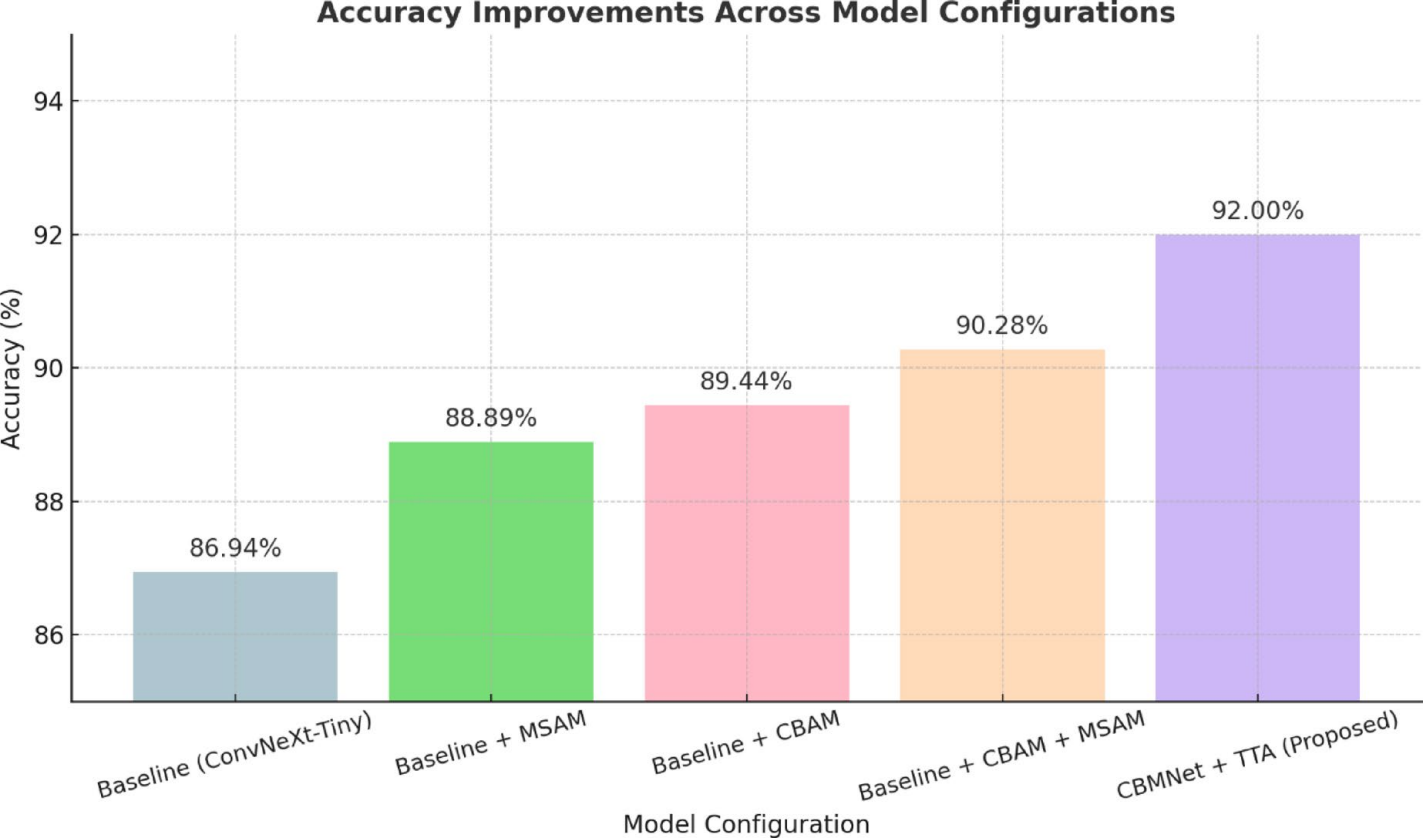
Accuracy comparison across different model variants evaluated in the ablation study.

Figure 16 depicts a clear upward trend in accuracy across successive configurations, highlighting the effectiveness of each architectural enhancement. The baseline model, despite its robust ConvNeXt-Tiny backbone, exhibits the lowest performance, clearly demonstrating that it alone cannot fully exploit the complex features necessary for accurate dental pathology classification. Introducing MSAM independently provides a notable boost by capturing multi-scale global context, essential for interpreting nuanced dental structures. Similarly, the CBAM module independently enhances feature discrimination through focused channel and spatial attention mechanisms. Combining CBAM and MSAM modules yields a synergistic improvement, confirming that simultaneous enhancement of global contextual awareness and localized spatial-channel attention is crucial. The final addition of TTA further elevates the model’s robustness and generalization capabilities, producing the best overall results. These ablation experiments conclusively validate our architectural decisions, highlighting the necessity and complementary roles of each module within the proposed CBMNet architecture.

**Fig. 17:**
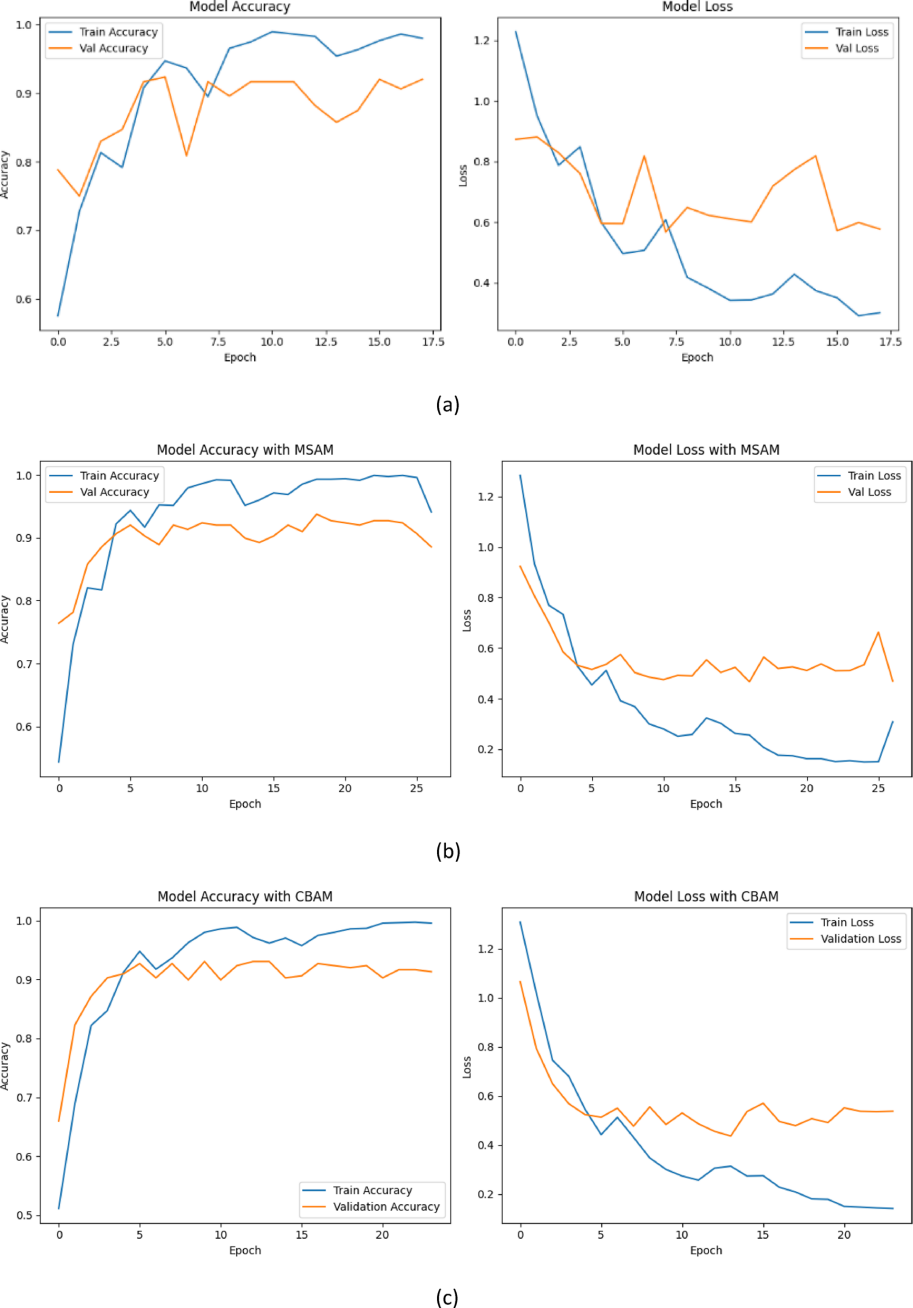

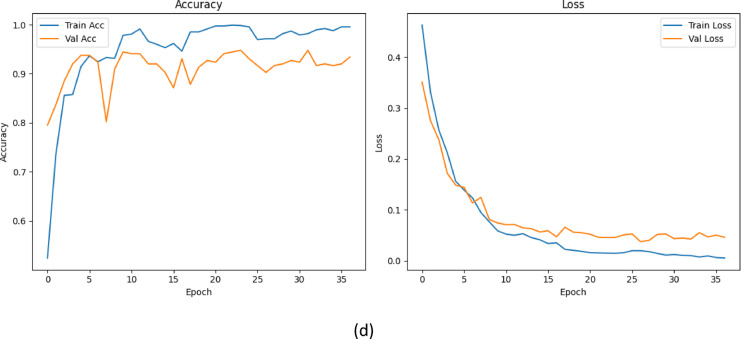
Training and validation accuracy/loss curves for different model variants. (a) Baseline (ConvNeXt-Tiny), (b) Baseline + MSAM, (c) Baseline + CBAM, (d) CBMNet (CBAM + MSAM).

All models are trained using the same hyperparameter settings and optimization schedules to ensure fairness, and the accuracy-loss curves for training and validation are illustrated in Fig. 17. In the baseline configuration, the model demonstrates a steady increase in training accuracy, approaching approximately 98% by the end of training. However, the validation accuracy plateaus early and fluctuates around 91%, while the validation loss remains relatively unstable beyond the mid-training epochs. Although the baseline model achieves acceptable performance, the divergence between training and validation curves suggests early signs of overfitting and suboptimal generalization. This behaviour likely stems from the model’s limited ability to attend to localized features essential for identifying subtle carious lesions.

The incorporation of MSAM in the second configuration yields a comparable improvement. The validation accuracy hovers around 93–94%, and the learning curves demonstrate enhanced stability compared to the baseline. MSAM’s design allows the model to aggregate contextual features across multiple scales, which is particularly beneficial in dental imaging where lesions vary in size and appearance. The smoother decline in validation loss and absence of significant fluctuations support the notion that MSAM contributes to more robust and context-aware feature extraction.

Upon introducing the CBAM module, we observe a marked improvement in generalization. Validation accuracy increases more rapidly and stabilizes near 93%, with a corresponding reduction in validation loss. The training accuracy also improves further, exceeding 99%. This outcome validates the effectiveness of CBAM in guiding the model’s attention toward semantically relevant spatial and channel-wise regions. By selectively enhancing discriminative features, CBAM aids the model in suppressing background noise and improving its focus on lesion-bearing areas in dental radiographs.

Finally, our proposed model, CBMNet, which combines both CBAM and MSAM, exhibits the most favourable learning dynamics. Training and validation accuracies consistently remain above 99% and 94%, respectively, while validation loss reaches its lowest value (0.06). The overlap of training and validation curves across both accuracy and loss further suggests minimal overfitting and strong generalization capabilities. This synergy between CBAM’s spatial-channel sensitivity and MSAM’s hierarchical context modelling results in a model that is not only highly accurate but also resilient to noise and anatomical variability.

**Fig. 18:**
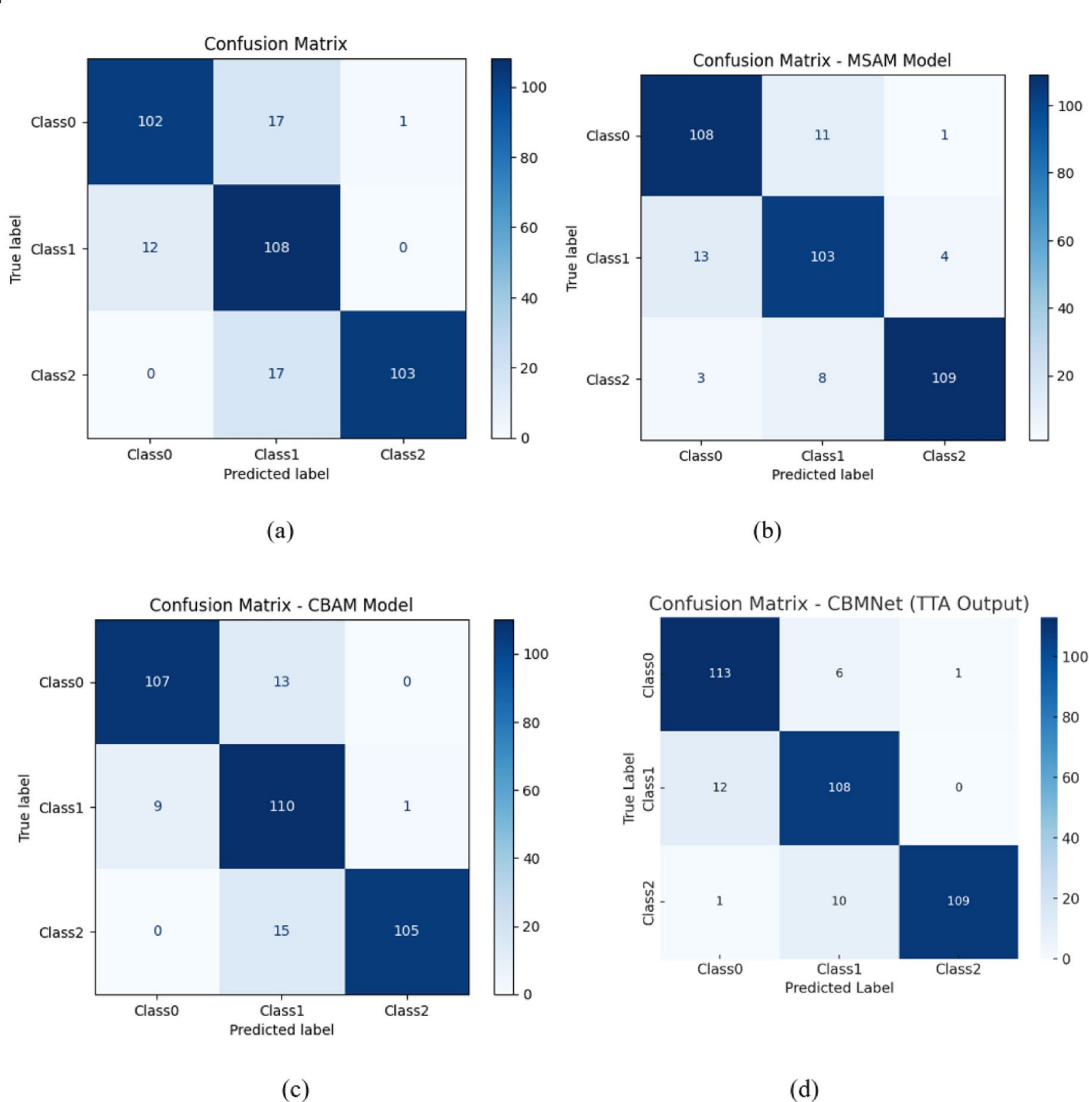
Confusion matrices for different model variants: (a) Baseline (ConvNeXt-Tiny), (b) Baseline + MSAM, (c) Baseline + CBAM, (d) CBMNet (CBAM + MSAM) + TTA.

To provide deeper insight into the class wise performance of each model configuration, we present confusion matrices corresponding to all five experimental variants. These matrices as shown in Fig. 18, illustrate how each architecture correctly and incorrectly classifies images across the three dental caries classes. These visualizations support our claim that each incremental module contributes to more balanced predictions across all classes, particularly improving sensitivity for underrepresented classes.

To statistically validate the ablation study, we compared CBMNet with its baseline and attention-only variants using both binomial confidence intervals and McNemar’s test on the held-out test set (*n* = 360). CBMNet achieved the highest performance with an accuracy of 91.7% (95% CI: 88.4–94.1%), outperforming the baseline ConvNeXt-Tiny (86.9%, 95% CI: 83.1–90.0%; *p* < 0.0001) and the MSAM-only variant (88.9%, 95% CI: 85.2–91.7%; *p* = 0.002) with statistical significance. While CBMNet also exceeded the CBAM-only model (89.4%, 95% CI: 85.8–92.2%) in accuracy, the improvement did not reach statistical significance (*p* = 0.057). Nevertheless, CBMNet consistently demonstrated higher precision, recall, and F1-scores than CBAM, and its accuracy interval lay above that of all ablated variants. These findings indicate that the dual-attention architecture provides statistically meaningful gains over the baseline and MSAM-only variants, while suggesting that a larger dataset may be required to establish significance over CBAM alone.

### Comparison with state-of-the-art models

Most of the recent research that have utilized deep learning to find and classify cavities in teeth have used panoramic radiography. ResNet50, EfficientNetB0, and DenseNet121 are some of the deep learning architectures that are often used in these kinds of investigations. Panoramic imaging displays a lot of the dental arches and the structures around them, but it doesn’t give enough information to accurately detect specific dental lesions, especially when they are still little or not very evident.

Our study, on the other hand, employs high-resolution periapical radiographs, which show more anatomical information and are better at spotting carious lesions that are only in one place. This makes the diagnosis more useful and accurate in a clinical setting. We compare the suggested CBMNet architecture to the deep learning models we discussed above (Table 8). These models have been shown to function effectively when you need panoramic images. We used our periapical radiography dataset to compare our CBMNet model to three of the finest models: ResNet50, EfficientNetB0, and DenseNet121. In the section "Experimental configuration", it specifies that all models are trained and tested using the same experimental settings, data splits, preprocessing procedures, augmentation methods, and hyperparameters.

**Table 9:** Comparison of test accuracy with and without Test-Time augmentation (TTA).

Evaluation mode	Test Accuracy
Plain Inference	91.11%
TTA (5 rounds)	91.67%

**Fig. 19:**
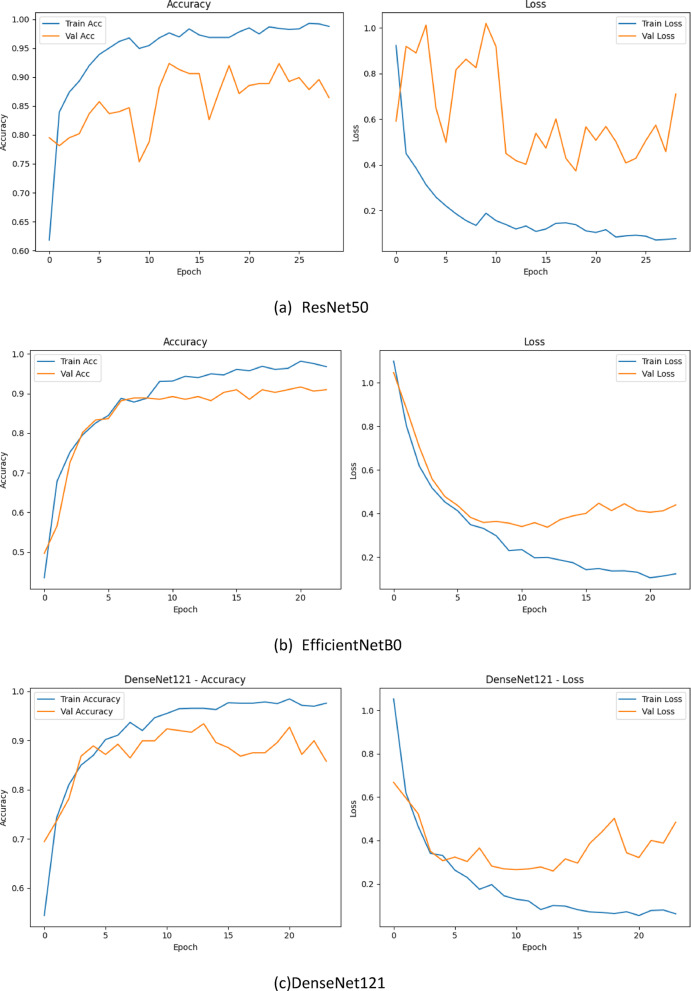

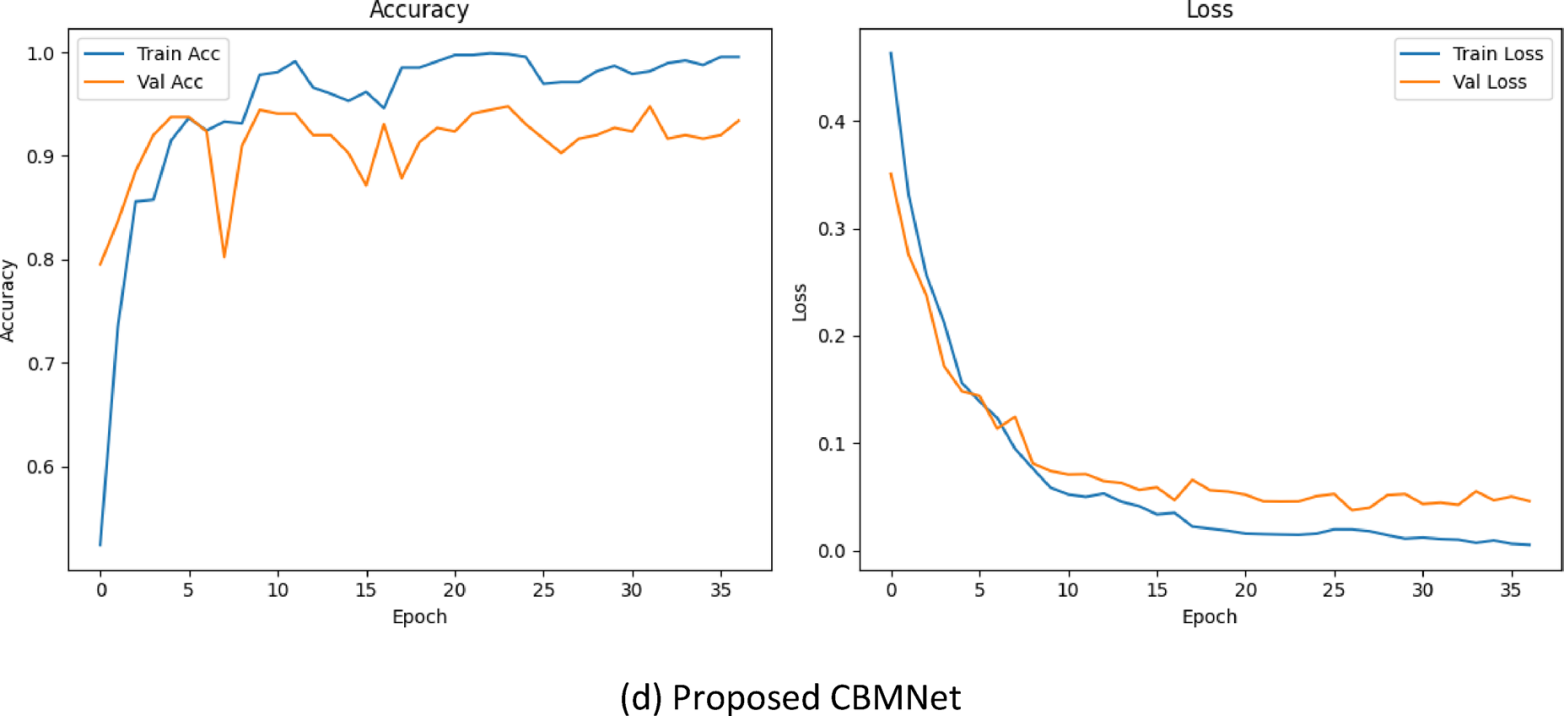
Accuracy and loss plots for different models.

**Fig. 20:**
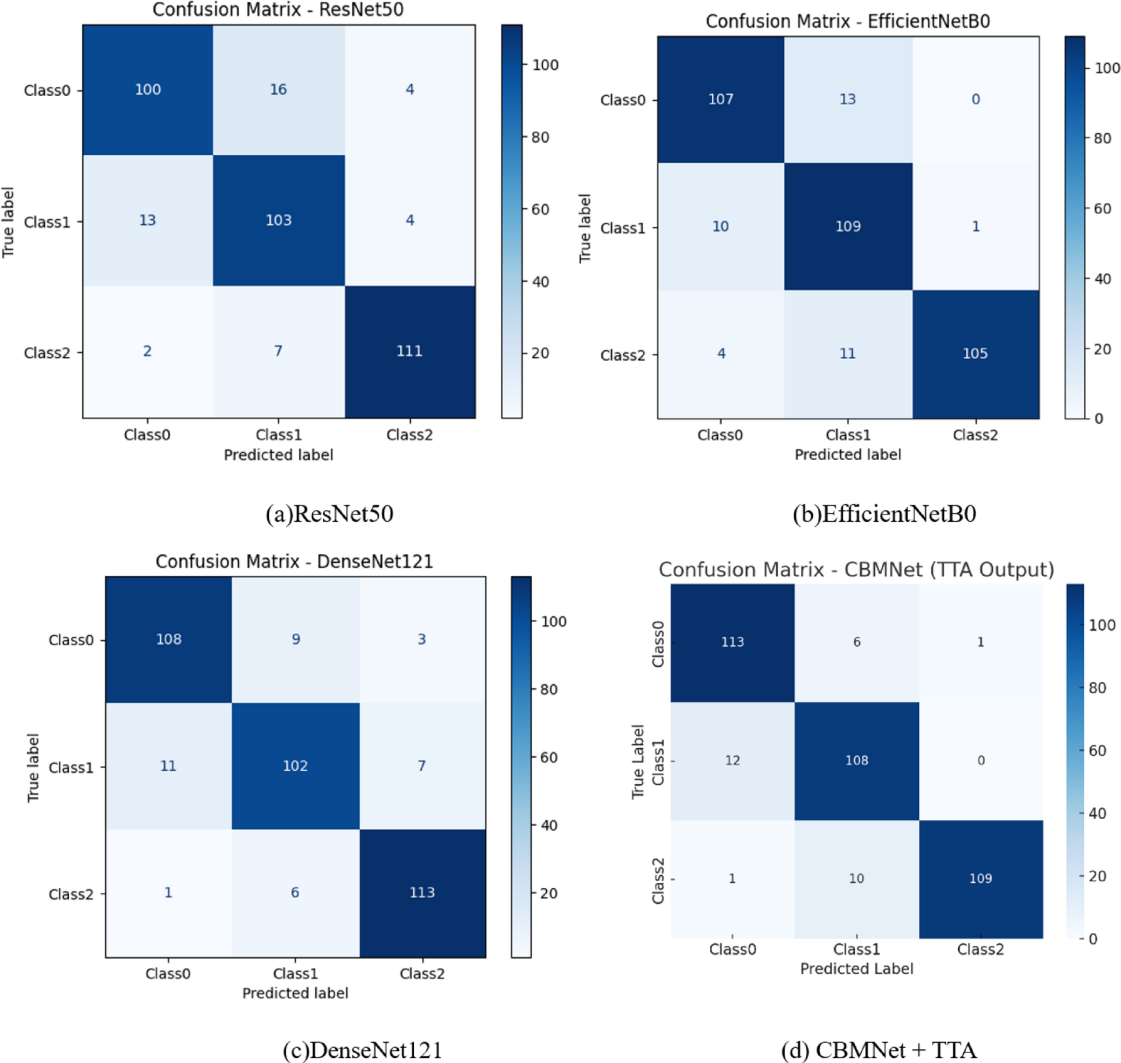
Confusion matrices for each model: (a) ResNet50, (b) EfficientNetB0, (c) DenseNet121, (d) CBMNet (CBAM + MSAM) + TTA.

Figure 19 displays the training and validation accuracy/loss curves for each model configuration, while Fig. 20 presents the corresponding confusion matrices. These visualizations collectively provide a comprehensive picture of both convergence behaviour and classification performance across classes.

ResNet50 : ResNet50 achieves smooth convergence in both accuracy and loss curves. However, the validation loss exhibits greater fluctuation compared to training loss, indicating minor overfitting. The confusion matrix highlights moderate class confusion between Class 0 and Class 1. Overall, ResNet50 achieves reliable performance, particularly for Class2, but its sensitivity for Class 1 is slightly lower than our proposed model.

EfficientNetB0 : The EfficientNetB0 model converges rapidly and demonstrates consistently high training and validation accuracy. Its confusion matrix reveals improved inter-class discrimination, with minimal false positives. Notably, the model shows balanced performance across all three classes, with superior precision for Class 1. This supports EfficientNetB0’s strength in modelling high-resolution dental features even with limited data.

DenseNet121: DenseNet121 exhibits highly stable convergence, and its validation loss curve remains closely aligned with the training loss curve. The confusion matrix confirms that DenseNet121 is particularly effective in minimizing false negatives for Class 2. However, mild confusion between Class 0 and Class 1 persists. Overall, DenseNet121 provides competitive generalization but lacks the attention-guided interpretability introduced in our architecture.

Proposed CBMNet : In contrast to the above models, CBMNet outperforms all SOTA base lines in both overall accuracy and class-specific recall. Our model converges more rapidly and achieves a validation accuracy that remains consistently high across epochs. The confusion matrix demonstrates a more even distribution of correct predictions and fewer inter-class confusions, validating the effectiveness of the integrated CBAM and MSAM attention modules.

### Final evaluation on held-out test set

After cross-validation, the model is retrained on the entire training set using the best hyperparameters. Evaluation is performed on the held-out test set(*n* = 360 samples), both under standard inference and using Test-Time Augmentation(TTA).

The use of TTA led to a marginal but meaningful improvement of + 0.56% (Table 9), suggesting better robustness to input variability(e.g., orientation, brightness shifts).

**Table 10:** Class-wise precision, recall, and F1-score for the proposed model.

Metric	Class1	Class2	Class3	Macro Avg
Precision	0.90	0.87	0.99	0.92
Recall	0.94	0.90	0.91	0.92
F1-Score	0.92	0.89	0.95	0.92
Support	120	120	120	

Precision = Positive Predictive Value; Recall = Sensitivity; F1-score = Harmonic mean of precision and recall.

### Detailed evaluation metrics

The classification report on the TTA-based predictions is shown below in Table 10.

**Table 11:** Distribution of False Positives (FP) and False Negatives (FN) in CBMNet Predictions (*n* = 360).

True Class	Misclassified as	Count	% of Total Predictions	Error Type	Clinical Implication
Class I	Class II	6	1.7%	False Positive	May lead to unnecessary restorative treatment
Class I	Class III	1	0.3%	False Positive	Severe overtreatment (rare)
Class II	Class I	12	3.3%	False Negative	Undertreatment; lesion progression risk
Class III	Class II	10	2.8%	False Negative	Undertreatment of advanced lesions
Class III	Class I	1	0.3%	False Negative	Extreme undertreatment (rare)

False positives (FP) are cases where a milder lesion (Class I) was predicted as more severe (Class II or III). False negatives (FN) are cases where a more severe lesion (Class II or III) was predicted as a milder class.

Each class exhibits strong per-class performance, with Class3 achieving the highest precision (0.99) and F1-score(0.95), indicating excellent model confidence in classifying this category. Class0 achieved the highest recall (0.94), ensuring that very few true cases are missed.

- Precision is high across all classes, particularly Class3, confirming that the model avoids false positives effectively.
- Recall remains consistently high (> 0.90) across all classes which is essential in clinic screening where missing a true case (false negative) is costly.
- F1-Score balances the trade-off between precision and recall and remains uniformly strong, indicating model consistency.
- Macro Average validates uniformity of model performance across classes.
- Weighted Average confirms that class imbalance did not bias the overall evaluation.

## Discussion

Recent studies have begun to explore ConvNeXt in medical imaging. For example, Liu et al.^39^ demonstrated its utility for periapical lesion detection when compared with ResNet34, and Jian Liu et al^40^ integrated ConvNeXt with object detection in their YoCNET framework for binary lesion classification. Beyond dental radiographs, ConvNeXt-inspired models have been investigated in segmentation tasks^41^ and in lightweight classification frameworks for general medical images^42^. While these works highlight ConvNeXt’s emerging role in healthcare applications, they are primarily limited to lesion detection or segmentation. To the best of our knowledge, the present study is one of the first to adapt ConvNeXt specifically for clinically interpretable, multi-class caries classification using G.V. Black categories in periapical radiographs. This distinction underscores the novelty of CBMNet, which extends beyond standard ConvNeXt applications by incorporating dual-attention modules, GAN-based augmentation for class balance, and PSO optimization for robust performance.

**Fig. 21:**
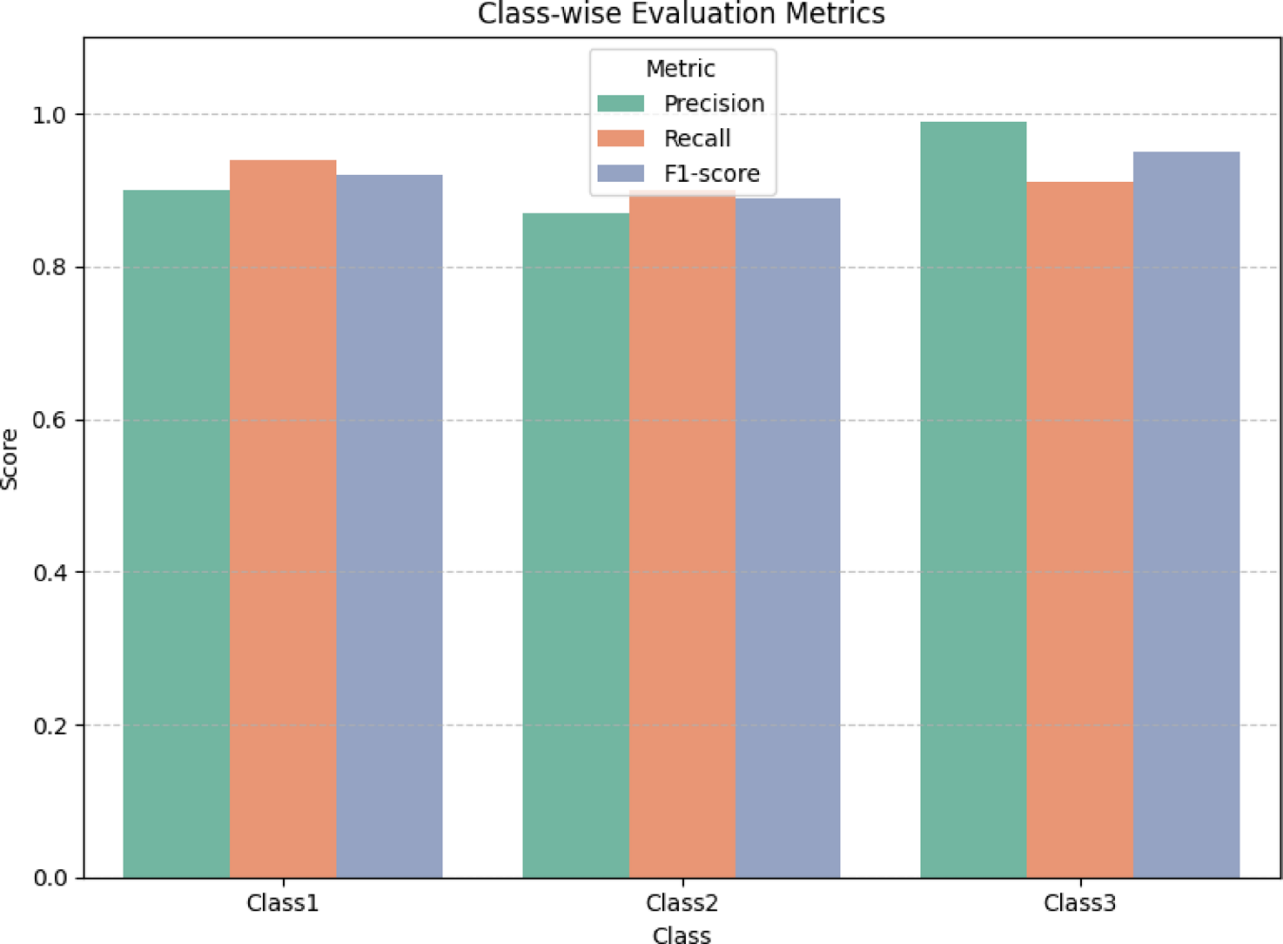
Bar plot of class-wise precision, recall, and F1 score.

CBMNet demonstrated robust performance in G.V. Black classification, achieving an overall test accuracy of 92% with precision, recall, and F1-scores exceeding 90% across all three classes. The class-wise evaluation metrics, including precision, recall, and F1-score, are visualized in Fig. 21 to highlight the model’s performance across different classes. This represents a clear improvement over baseline CNNs tested in our study, such as DenseNet121 (89% accuracy), EfficientNetB0 (89%), and ResNet50 (87%). While these architectures have previously been applied to dental imaging tasks with notable success, they often struggle with feature generalization when trained on small or imbalanced datasets. By integrating dual-attention mechanisms (CBAM and MSAM), CBMNet enhances feature localization in subtle carious regions, mitigating the generalization limitations of earlier CNN-based approaches. Our findings also align with and extend prior reports that highlighted the promise of deep learning for dental radiographs. For example, studies employing EfficientNet or ResNet variants for caries detection reported strong performance but noted challenges in handling underrepresented lesion classes and in scaling across diverse populations. CBMNet addresses these issues through StyleGAN2-ADA augmentation to balance rare lesion types and PSO-based optimization to stabilize training.

In the context of this study, false negatives were defined as cases where a more severe lesion (Class II or III) was underestimated as a milder class, while false positives were defined as cases where a milder lesion (Class I) was overestimated as a more severe class. This distinction is clinically important: false negatives risk undertreatment and disease progression, whereas false positives risk unnecessary restorative intervention and removal of healthy tooth structure.

Analysis of the confusion matrix (Fig. 18d) showed that CBMNet achieved correct predictions in 91.7% of all test cases, with relatively few misclassifications. Across the 360 samples, false negatives accounted for only 6.4% of total predictions, mainly involving Class II lesions misclassified as Class I (3.3%) and Class III lesions misclassified as Class II (2.8%). False positives were even rarer, occurring in only 1.9% of predictions, with most cases being Class I lesions misclassified as Class II (1.7%). Extreme cross-category errors (Class I ↔ Class III) were very uncommon (< 1% of all predictions), minimizing the risk of clinically severe mismanagement. These distributions of false positives and false negatives, along with their clinical implications, are summarized in Table 11.

Beyond CNN-based caries detection, alternative deep learning strategies have been successfully introduced in dentistry. For instance, transformer and hybrid models have recently been applied to panoramic radiographs and cone-beam CT, while AI approaches have been extended to domains such as skeletal maturation assessment^43^.These examples highlight how AI is reshaping multiple areas of dental diagnostics and treatment planning. By situating CBMNet within this broader landscape, we demonstrate that our framework not only improves upon CNN-based baselines for caries classification but also contributes to the wider evolution of dental AI methodologies aimed at improving consistency, early detection, and clinical reliability.

This study contributes to the growing intersection of artificial intelligence and clinical dentistry by addressing a critical need: reliable, automated detection of dental caries in radiographs. Current practice heavily relies on expert interpretation, which can be time-consuming, subjective, and prone to inter-observer variability. By achieving an accuracy of 92% on real-world clinical data, the proposed model surpasses traditional machine learning baselines and offers a decision-support tool for clinicians. Its application can reduce diagnostic time, assist in early-stage detection of lesions, and enhance consistency in patient management. A key factor underlying CBMNet’s performance is the dual-attention mechanism (CBAM + MSAM). By combining channel and spatial weighting, CBAM highlights diagnostically relevant features, while MSAM aggregates information across multiple receptive field sizes. Together, these modules enhance the network’s sensitivity to small or low-contrast lesions that might otherwise be missed, particularly in anterior proximal surfaces where radiographic detection is most challenging. This attention-guided approach ensures that the model prioritizes true anatomical patterns of caries rather than spurious texture cues or dataset biases – a critical property for trustworthy medical AI.

While our results are promising, several limitations need to be acknowledged. First, although StyleGAN2-ADA augmentation helped reduce class imbalance, the overall dataset was relatively small and was collected from a single institution. This means the model may not fully capture the variety of radiographs produced by different X-ray machines or clinical settings. Second, all patients were adults between 18 and 65 years of age. Younger children and older adults, who often present with different dental structures and radiographic patterns, were not represented in this study.

Another limitation is the unequal distribution across G.V. Black classes. Class III lesions were less common and therefore supplemented more heavily with synthetic images. Even though these images were screened for quality and realism, they may not perfectly reflect the true lesion anatomy. The model was also trained only on periapical radiographs, so its performance on other modalities such as bitewing or panoramic images is still unknown. Finally, although we used CBAM and MSAM to improve lesion localization, we did not visualize the attention maps in this work, which limits insight into exactly which features guided the network’s decisions.

In future, we aim to address these issues by gathering data from multiple centres, including wider age groups and balancing lesion types with real cases rather than synthetic ones. We also plan to visualize attention maps and compare model outputs directly with clinician performance, which will strengthen interpretability and clinical trust.

Although our model achieved an encouraging test accuracy of 92%, there remains ample scope for further improvement. One avenue of exploration is transitioning from convolution-based architectures to transformer-based models. Vision Transformers (ViTs) and hybrid CNN–Transformer architectures have shown strong potential in medical imaging by modelling long-range dependencies and integrating global contextual features. We also plan to incorporate interpretability techniques such as Grad-CAM and attention heatmaps, which can help clinicians visualize the regions driving model decisions and thereby build confidence in AI-assisted diagnosis.

Beyond these technical advances, future work should address clinical integration and validation at scale. Expanding to multi-centre datasets with broader demographic diversity and varied imaging systems will be essential for establishing generalizability. Incorporating multi-modal data (radiographs combined with clinical notes or demographic factors) and semi-supervised learning approaches may further strengthen robustness while reducing dependence on manual annotations. Finally, translating CBMNet into real-time chairside applications has the potential to support dentists directly during clinical workflows, reducing diagnostic time and improving consistency. Collectively, these directions move beyond algorithmic refinement toward ensuring CBMNet’s true translational impact in everyday dental practice.

## Conclusion

This study introduced CBMNet, an attention-enhanced deep learning framework for the automated classification of dental caries in intraoral periapical radiographs. By integrating CBAM and MSAM within a ConvNeXt-Tiny backbone and optimizing hyperparameters through PSO, CBMNet demonstrated superior diagnostic accuracy (92%) compared with state-of-the-art CNN baselines such as DenseNet, EfficientNet, and ResNet, highlighting the effectiveness of dual-attention in enhancing lesion localization. Beyond technical gains, CBMNet has direct clinical relevance. Its ability to detect subtle and low-contrast lesions supports earlier and more consistent diagnosis, reducing the risk of missed or delayed treatment. As a decision-support tool, it could be particularly valuable in assisting less experienced clinicians, standardizing diagnostic outcomes, and reducing inter-observer variability. By addressing class imbalance, enhancing interpretability through attention mechanisms, and validating performance with rigorous cross-validation and test-time augmentation, CBMNet represents a practical step toward AI-assisted dentistry. Future work will extend to multi-centre datasets, transformer-based models, and real-time chairside applications to further establish generalizability and translational impact.

## Data Availability

The clinical image data collected from Sibar Institute of Dental Sciences, Guntur are confidential and are not publicly available due to institutional policies that restrict sharing with third parties. The institute has not granted permission to share the original patient radiographs. However, the synthetic radiographs generated using the StyleGAN2-ADA model during the current study can be shared. These datasets are available from the corresponding author upon reasonable request. To enhance transparency and reproducibility, all codes are available at CBMNet-GVBlack.git and archived with a DOI at [https://doi.org/10.5281/zenodo.16436223].
